# Integration of genetic and metabolic features related to sialic acid metabolism distinguishes human breast cell subtypes

**DOI:** 10.1371/journal.pone.0195812

**Published:** 2018-05-30

**Authors:** Christopher T. Saeui, Alison V. Nairn, Melina Galizzi, Christopher Douville, Prateek Gowda, Marian Park, Vrinda Dharmarha, Sagar R. Shah, Amelia Clarke, Melissa Austin, Kelley W. Moremen, Kevin J. Yarema

**Affiliations:** 1 Department of Biomedical Engineering and the Translational Tissue Engineering Center, The Johns Hopkins University, Baltimore, Maryland, United States of America; 2 Complex Carbohydrate Research Center, University of Georgia, Athens, Georgia, United States of America; 3 Department of Biochemistry and Molecular Biology, University of Georgia, Athens, Georgia, United States of America; National Cancer Institute at Frederick, UNITED STATES

## Abstract

In this report we use ‘high-flux’ tributanoyl-modified *N*-acetylmannosamine (ManNAc) analogs with natural N-acetyl as well as non-natural azido- and alkyne N-acyl groups (specifically, 1,3,4-O-Bu_3_ManNAc, 1,3,4-O-Bu_3_ManNAz, and 1,3,4-O-Bu_3_ManNAl respectively) to probe intracellular sialic acid metabolism in the near-normal MCF10A human breast cell line in comparison with earlier stage T-47D and more advanced stage MDA-MB-231 breast cancer lines. An integrated view of sialic acid metabolism was gained by measuring intracellular sialic acid production in tandem with transcriptional profiling of genes linked to sialic acid metabolism. The transcriptional profiling showed several differences between the three lines in the absence of ManNAc analog supplementation that helps explain the different sialoglycan profiles naturally associated with cancer. Only minor changes in mRNA transcript levels occurred upon exposure to the compounds confirming that metabolic flux alone can be a key determinant of sialoglycoconjugate display in breast cancer cells; this result complements the well-established role of genetic control (e.g., the transcription of STs) of sialylation abnormalities ubiquitously associated with cancer. A notable result was that the different cell lines produced significantly different levels of sialic acid upon exogenous ManNAc supplementation, indicating that feedback inhibition of UDP-GlcNAc 2-epimerase/ManNAc kinase (GNE)—generally regarded as the ‘gatekeeper’ enzyme for titering flux into sialic acid biosynthesis—is not the only regulatory mechanism that limits production of this sugar. A notable aspect of our metabolic glycoengineering approach is its ability to discriminate cell subtype based on intracellular metabolism by illuminating otherwise hidden cell type-specific features. We believe that this strategy combined with multi-dimensional analysis of sialic acid metabolism will ultimately provide novel insights into breast cancer subtypes and provide a foundation for new methods of diagnosis.

## Introduction

Sialic acids were discovered over eighty years ago by Swedish chemist Gunnar Blix [1]; it is now known that these carboxylic acid containing, nine carbon monosaccharides ubiquitously decorate the outer termini of glycans found on animal cells and are well-recognized to play important roles in human development, health, and disease. To give three examples: knockout of genes involved in sialic acid metabolism causes severe developmental defects in rodent models and can be embryonically lethal [2–4]; sialylation of biomolecules such as immunoglobulins and hormones affects their half-lives, bioactivities, and distribution throughout the body [5–9]; and sialic acids play critical roles in the brain related to learning, neural plasticity, memory, and development [10–12]. An outstanding example of abnormal sialylation in disease is the near-universal overexpression of sialic acids in cancer [13–15], which drives multiple aspects of oncogenesis including disabling immunesurveillance by tempering immunity via the sialic acid binding immunoglobulin like lectin (SIGLEC) immunomodulatory signaling pathway [16–18]; facilitating metastasis [14,19,20]; and potentiating oncogenic surface receptors such as EGFR [21–23].

Intervention in sialylation to develop therapies for diseases characterized by aberrant sialic acid metabolism has been difficult to achieve. One promising approach to manipulate sialylation, however, is based on ‘metabolic glycoengineering’ (MGE [24], also known as ‘metabolic oligosaccharide engineering’ [25,26]) where exogenously-supplied monosaccharide analogs co-opt the metabolic and biosynthetic machinery of a cell that builds glycans. Nascent MGE efforts were first reported ~35 years ago with the serendipitous discovery that fluorinated analogs of ManNAc designed to inhibit the biosynthetic overproduction of sialic acid in cancer instead were unexpectedly incorporated into cell surface glycans [27,28]. Werner Reutter and colleagues deliberately exploited this substrate promiscuity to install non-natural sialosides with extended alkyl N-acyl groups into glycans *in vivo* [29,30] and Bertozzi and colleagues pioneered the incorporation of bio-orthogonal chemical functional groups (e.g., ketones [31] and azides [32]) into glycans using MGE. Since then, analog diversity has continued to expand (25 or more non-natural different N-acyl groups can be accommodated by the sialic acid biosynthetic machinery [33]) and practical applications of MGE (e.g., for the treatment of disease) have been pursued, as outlined in reviews by our team [24,26,33] and others [25,34].

**Fig 1:**
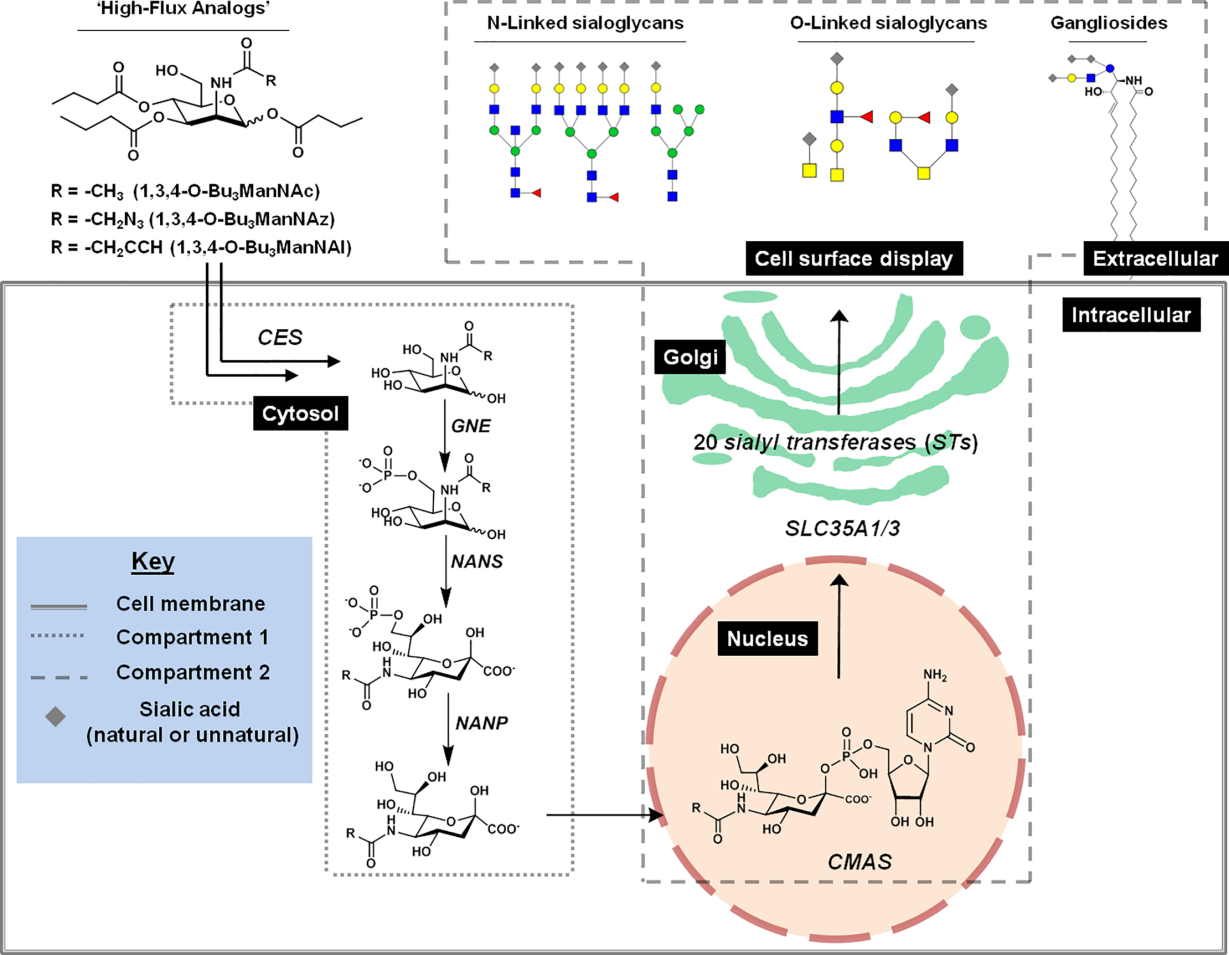
Overview of ManNAc analog metabolism *via* sialic acid metabolism and glycosylation (SAMG) gene activity. ‘High-flux’ ManNAc analogs (1,3,4-O-Bu_3_ManNAc, 1,3,4-O-Bu_3_ManNAz, 1,3,4-O-Bu_3_ManNAl analogs) passively diffuse across the plasma membrane after which the core natural or R-modified ManNAc (i.e., ManNAc, ManNAz, or ManNAl) is released *via* non-specific carboxylesterases (*CES*) that hydrolyze the butyrate groups. ManNAc(R) is converted to sialic acids by the kinase activity of *GNE* and subsequent activities of *NANS*, and *NANP* in the cytosol; in this study these metabolites constitute ‘Compartment 1’ and are measured in aggregate using the periodate resorcinol assay. Once synthesized and dephosphorylated, sialic acid enters the nucleus where it is converted to the corresponding nucleotide sugar (e.g., CMP-Neu5Ac, CMP-Sia5Az, or CMP-Sia5Al) by *CMAS*; these activated sialic acids are then transported into the Golgi apparatus by *SLC35A1* and *SLC35A3* where a subset of the 20 human sialyltransferases created sialoglycoconjugates (primarily, N- and O- linked glyocoproteins or gangliosides [i.e., sialic acid-modified glycosphingolipids]) and these compounds constitute ‘Compartment 2’ and are also measured in aggregate using the periodate resorcinol assay (as outlined in the Materials and Methods section).

One shortcoming of MGE is the low efficiency of hexosamine analog utilization by cells. To remedy this difficulty, attempts to increase cellular uptake of ManNAc analogs (and other mono- and disaccharides) were pursued using a peracetylation strategy that masks a sugar’s hydroxyl groups and thus increases uptake by facilitating plasma membrane diffusion [35–37]. Unfortunately this strategy often results in moderate, but nevertheless unacceptable, growth inhibition and even cytotoxicity [38,39]. To overcome these limitations, we designed partly acylated monosaccharides with a ‘1,3,4’ substitution pattern that masks three of the four hydroxyl groups of a hexosamine with the longer short chain fatty acid (SCFA) butyrate [40,41]. These triacylated analogs, exemplified by 1,3,4-O-Bu_3_ManNAc (Fig 1), compensate for the loss of masking of one of the hydroxyl groups that renders triacetylated analogs (e.g., 1,3,4-O-Ac_3_ManNAc) membrane impermeable through the increased lipopholicity of butyrate compared to acetate (the physicochemical properties of these analogs are described in detail in a recent publication [42]). Most critically, this strategy sidesteps growth inhibition, cytotoxicity, and a suite of ‘off-target’ effects found in C6OH ester modified hexosamines [40,43–47].

In previous studies we showed that 1,3,4-O-Bu_3_ManNAc, which we call a ‘high-flux’ analog because of its ability to substantially enhance sialylation at concentrations where off-target effects such as altered global transcription [43,45] are minimized [40,41], can selectively increase the natural sialylation (i.e., Neu5Ac levels) of specific glycoproteins in cancer cells [47]. To provide context for this discovery, the first ~25 years of MGE largely focused on the replacement of natural glycans on the cell surface with their non-natural counterparts with only a few reports [39,48] devoted to examining intracellular metabolism (e.g., flux through the relevant nucleotide sugar biosynthetic pathways). The need to more thoroughly evaluate metabolism in MGE analog-treated cells has become increasingly compelling, however, as metabolic profiling gains increasing promise for diverse purposes ranging from evaluating stem cell pluripotency [49–51], monitoring diabetes [52,53], to characterizing cellular physiology in cancer [54–56]. In an example related to cancer, glycolytic flux and glucose metabolism influenced sialylation in malignant breast cells [57,58], leading us to query whether our high-flux ManNAc analogs could be used to perturb sialic acid biosynthesis in unique, cell type-dependent manners and thereby provide insights into this type of cancer.

Accordingly, we employed a trio of ManNAc analogs (Fig 1) that either increase levels of natural sialic acid (i.e., 1,3,4-O-Bu_3_ManNAc) or replace natural metabolites with their chemically-altered counterparts (e.g., azido- or alkyne-modified sialosides derived from 1,3,4-O-Bu_3_ManNAz or 1,3,4-O-Bu_3_ManNAl, respectively) to probe the sialic acid metabolism of breast cell lines. Our study also included a detailed evaluation of the transcription of the relevant ‘glyco-genes’ and surveyed the impact of the analogs on sialoglycoconjugate levels. The resulting data revealed physiological differences and metabolic capabilities that are unique to each cell line that are hidden in the absence of analog supplementation, thereby highlighting the usefulness of MGE for understanding biochemical pathway function in a novel way that ultimately may provide a new approach for cancer biomarker discovery and diagnosis.

## Materials and methods

### Materials

All chemicals and materials required for analog synthesis were purchased from Sigma Aldrich (St. Louis, MO). The cell lines MCF10A (ATCC CRL-10317), T-47D (ATCC HTB-133), and MDA-MB-231 (ATCC CRM-HTB-26) were purchased from American Type Culture Collection (ATCC, Manassas, VA). Before conducting cell experiments, each cell line was authenticated through the Johns Hopkins Genetic Resources Core Facility using short tandem repeat (STR) profiling according to the National Institutes of Health (NIH) recommendations. Each cell line’s STR data was also cross-referenced with both the ATCC and the German Collection of Microorganisms and Cell Cultures (DSMZ) data repositories for authentication. N-Acetyl-D-mannosamine (ManNAc) analogs 1,3,4-O-Bu_3_ManNAc and 1,3,4-O-Bu_3_ManNAz were synthesized as previously described [40,46]. The synthesis and characterization of the alkyne modified analog 1,3,4-O-Bu_3_ManAl, a previously unreported compound, is provided in the Supporting Information (S1 Fig).

### Cell culture

The T-47D and MDA-MB-231 cell lines were maintained routinely in RPMI 1640 medium (Corning 10-040-CV) supplemented with 10% (v/v) fetal bovine serum (FBS, Corning 35-011-CV) and the appropriate dilution of 20x antibiotic-antimycotic solution (Thermofisher 15240062). MCF10A cells were maintained routinely in the same media supplemented with 10 μg/mL insulin (Thermofisher 12585014) and 5.0 μg/mL hydrocortisone (Sigma H0888); these media are referred as ‘growth media’ below. RPMI 1640 medium (Corning 10-040-CV). As noted below, sialic acid metabolism and quantification experiments were conducted with reduced FBS (1.0% v/v) in the absence of antibiotics-antimycotics (to avoid sialyltransferase inhibition and to reduce recycling of sialic acid containing serum components [57,59,60]); this medium is referred to as ‘assay media’ below. It might be noted that aside from these two modifications (i.e., low FBS and no antibiotics) standard cell culture conditions optimized for each cell line for routine cell culture (e.g., media type, glucose concentration, and initial cell densities) were used during the analog supplementation assays (note that we avoid extreme deviations from ‘normal’ culture conditions, which can have a major impact on sialic acid metabolism [57,58]). A long-standing feature of ManNAc-based sialic acid glycoengineering experiments has been the ability to deploy this technique across a variety of conditions (e.g., different initial cell seeding densities or ‘high’ vs. ‘low’ glucose concentrations) with the most important determinant of successful analog metabolism being the maintenance of cell viability [38,39,61], which we measure below (and show no adverse analog-induced impact).

### Cell proliferation assays

Cells cultured in growth media were collected via trypsinization, counted, and plated in 150 mm tissue culture dishes (5.0 x 10^6^ cells per dish) in 20 mL of assay medium. The cells were allowed to attach to the plates overnight, and then treated with 1,3,4-O-Bu_3_ManNAc, 1,3,4-O-Bu_3_ManNAz, or 1,3,4-O-Bu_3_ManNAl (0, 10, 100 and 250 μM) by adding appropriate dilutions of analog from a stock solution of 100 mM maintained in ethanol. Four replicates for three time points (6, 24, and 48 h) were tested for each of the three analogs. The untreated controls were exposed to the equivalent volume of ethanol given to cells subject to the 250 μM dose. At the specified time points (6, 24, and 48 h), the cells were detached using non-enzymatic buffer (Cellstripper Corning 25-056-CI) and cell counts were performed as previously described [46] using a Beckman-Coulter Z2 Coulter Counter. Each experiment was performed in triplicate and cell counts were normalized to the control based on the six hour time point.

### Transcript analysis

Cells were plated and treated individually with one of the three analogs at 0 or 100 μM in a manner similar to the flow cytometry assays but using 100 mm tissue culture plates and 3.0 x 10^6^ cells per dish. After incubation with the analogs for 24 h, the cells were harvested by scraping, counted, and portioned into aliquots of 1.0 x 10^6^ cells from each line and treatment condition that were flash-frozen in liquid nitrogen and stored at -80°C until analysis. Total RNA isolation and cDNA synthesis on three biological replicates of each cell type and treatment condition was carried out as described previously in preparation for quantitative RT-PCR analysis of known human sialic acid metabolism and sialyltransferase (SAMG) genes [62]. The qRT-PCR reactions were performed in triplicate for each gene analyzed using primer pairs listed in the Supporting Information (S1 Table). Amplification conditions and data analysis was performed as described [63]; briefly, Ct values for each gene were normalized with the control gene, *RPL4*, prior to calculation of relative transcript abundance. Each experiment and PCR analysis was performed in triplicate. Statistical analyses were conducted for pairs of samples as well as multiple sample and treatment comparisons (Tukey’s test).

### Flow cytometry assays

Cells (5.0 x 10^5^ in 2.0 mL media) were placed into 6-well plates in assay media and treated with either 1,3,4-O-Bu_3_ManNAz or 1,3,4-O-Bu_3_ManNAl at 0 or 250 μM and incubated for 24 h. After 24 h, the assay medium was removed by aspiration and the cells were washed with PBS and harvested via scraping and collected in PBS. The cells were pelleted, fixed with 4% PFA in PBS for 10 min, and were washed with 2% BSA in PBS. The complementary Alexa Fluor 488 alkyne and azido probes (ThermoFisher A10267 and A10266) were conjugated to the cells using bioorthogonal ‘click’ chemistry; specifically, azido analog-treated cells were treated with biotin-PEG4-alkyne (Sigma 764213) and alkyne analog-treated cells were treated with azide-PEG3-biotin (Sigma 762024). Ascorbic acid (100 μL of a 500 mM stock) was added to 807 μL of PBS followed by 90 μL of copper (II) sulfate (11.1 mM stock). The labeling reaction was designed to minimize detection differences of the alkyne- and azide-modified sialic acids; for example we used copper catalyzed click ligation reactions in both cases that are not significantly affected by the steric and electronic properties of the groups attached to the alkyne and azido centers [64] instead of using more-efficient copper-free, ring strain-promoted ligation reactions available only available for labeling of azide-modified glycans [65]. The click reactions were mixed by rotating the tubes end over end every 10 min for a total of 1.0 h at room temperature. After labeling the cells were pelleted, washed 3x with 2% BSA in PBS and resuspended in 100 μL of 2% BSA in PBS. Quantitative analysis of cell labeling was performed using a BD Accuri C6 cytometer with FL2 optical detection. Each experiment was performed in triplicate (biological replicates).

### Periodate resorcinol quantification of sialic acid

Briefly, cells from each line were plated in 150 mm dishes and cultured in assay medium. Each analog was tested individually at concentrations of 0, 10, 100, and 250 μM for 6, 24, and 48 h. The untreated (0 μM) controls were given ethanol equal to the amount of this solvent vehicle as used for the highest dose (250 μM). After the specified times, each plate was aspirated, washed 3x with PBS, and the cells were detached with scraping and collected in PBS. The cells were counted after thorough resuspension and approximately 4.0 x 10^5^ to 1.5 x 10^6^ cells were used per sample (exact cell counts were recorded for each sample and used to normalize the sialic acid data to a ‘per cell’ basis as reported in the Results section). Quantification of the free and conjugate bound forms of sialic acid was performed using the established periodate resorcinol assay [66] (adapted to 96 well plate format [67]) using an 8-point standard curve. Briefly, this assay measures total sialic acid (i.e., all chemical species of sialic acids) by performing the periodate oxidation step under mild conditions (i.e., 10 min on ice). In parallel aliquots of each sample oxidized at 100°C for 90 min provides a measure of glycoconjugate-bound sialosides (which comprise ‘Compartment 2’ in this study). Compartment 1 levels of sialic acid (i.e., unconjuagated, free monosaccharide species) are measured by simple substraction of glycoconjugate-bound signal from the levels of total sialic acid. Of note, signal from this colorimetric assay is derived from the C7-C9 polyol chain of sialic acids [68,69] and is not measurably (i.e., ≥ 5%) affected by chemical alterations made to the N-acyl position of sialic acids (i.e., the “NAz” and “NAl” groups in this study. The amount of sialic acid determined from optical density (OD) readings and the standard curve was then normalized based on the number of cells in each sample. For each sample the parameter we define as ‘Reserve Capacity’ was calculated by taking the maximal amount of sialic acid measured at any time point or analog concentration and subtracting it from the basal amount of sialic acid measured in untreated control cells at the 6 h time point. Finally, sialic acid levels were plotted as a function of dose and time for each analog in each cell line and a surface was fit to the data using cubic spline interpolation (cubicinterp) in MATLAB. Briefly, cubic spline interpolation generates a piecewise continuous curve that passes through each values in a data set, providing a 3D surface representation of the kinetic- and dose-response of each cell line to each analog. Each set of time course of experiments where sialic acid production was monitored by periodate-resorcinol analysis was repeated in triplicate.

### Multiple regression modeling

A multiple regression model was developed to provide a mathematical basis for distinguishing cell subtype based on the parameters related to sialic acid metabolism described above based on the following general formula:

y=Xβ+ε

Briefly, X is a vector of independent variables (i.e., various sets of data obtained experimentally above), β is a vector of ‘coefficients’ (which are specifically defined below), and ε captures general ‘error,’ and y is the vector representing cell type with y = 1 for MCF10A, y = 2 for T-47D, and y = 3 for MDA-MB-231 cell types. A more detailed linear regression equation can be written in the general form as

$$y=\beta_{0}*1+\beta_{1}*x_{i1}+\beta_{2}*x_{i2}+\cdots +\beta_{ip}*x_{ip}+\varepsilon$$

where *x_ip_* = *value for feature p for sample i* and *βp = the regression coefficient for feature p*. The features used in our model include four genes (*x*_*i1*_ *= CMAS*, *x*_*i2*_ *= SLC35A3*, *x*_*i3*_ *= SLC35A1*, *and x*_*i4*_ *= NANP*) and three metabolism-based measurements based on ‘Reserve Capacity determined from supplementation with 1,3,4-O-Bu_3_ManNAc (*x*_*i5*_), 1,3,4-O-Bu_3_ManNAz (*x*_*i6*_), and 1,3,4-O-Bu_3_ManNAl (*x*_*i7*_). The specific data used to train the regression model is supplied in the S1 File (Supporting Information). The model was trained using the standard lm function in R (https://www.rstudio.com/) and used to generate regression coefficients for each feature and intercept values. Finally, the model was implemented for F-testing to assess overall significance.

## Results

### Cell growth and viability

The metabolic substrate-based studies described in this paper depend on the ability to introduce high levels of flux into the sialic acid biosynthetic pathway with negligible cytotoxicity and minimal growth inhibition. We previously achieved these objectives in other human (and rodent) cells using ‘high-flux’ 1,3,4-O-butanoylated ManNAc analogs [40,46,70,71] but the cytotoxicity of the newly-synthesized, alkyne-modified analog 1,3,4-O-Bu_3_ManAl was unknown; moreover, the three cell lines used in this study (MCF10A, T-47D and MDA-MB-231) had not been comparatively screened with these analogs in ‘assay media’ where the cells were grown in low serum (1.0%; to prevent recycling of sialic acids from glycoproteins contained in serum [57]) as well as without antibiotics (to avoid inhibition of sialylation [59]). Therefore, as a prelude to subsequent metabolic profiling, we first evaluated cell viability by measuring proliferation after treatment with 0, 10, 100, or 250 μM of 1,3,4-O-Bu_3_ManNAc (Panel A in S2 Fig), 1,3,4-O-Bu_3_ManNAz (Panel B in S2 Fig), or 1,3,4-O-Bu_3_ManNAl (Panel C in S2 Fig) for 6, 24, or 48 h and observed no statistically-significant differences between the control and test cells. These results indicated that these analogs—as expected based on our previous experience with ‘1,3,4’ tributanoylated hexomines over the concentration range we used—had no measurable effects on cell viability at concentrations needed to maximize sialic acid production and thus avoided the detrimental impact of fully acylated ManNAc analogs (e.g., Ac_4_ManNAc or Bu_4_ManNAc) on sialylation [38,39,43,61].

### Transcript analysis of sialic acid metabolism and glycosylation (SAMG) genes in untreated cells

**Fig 2:**
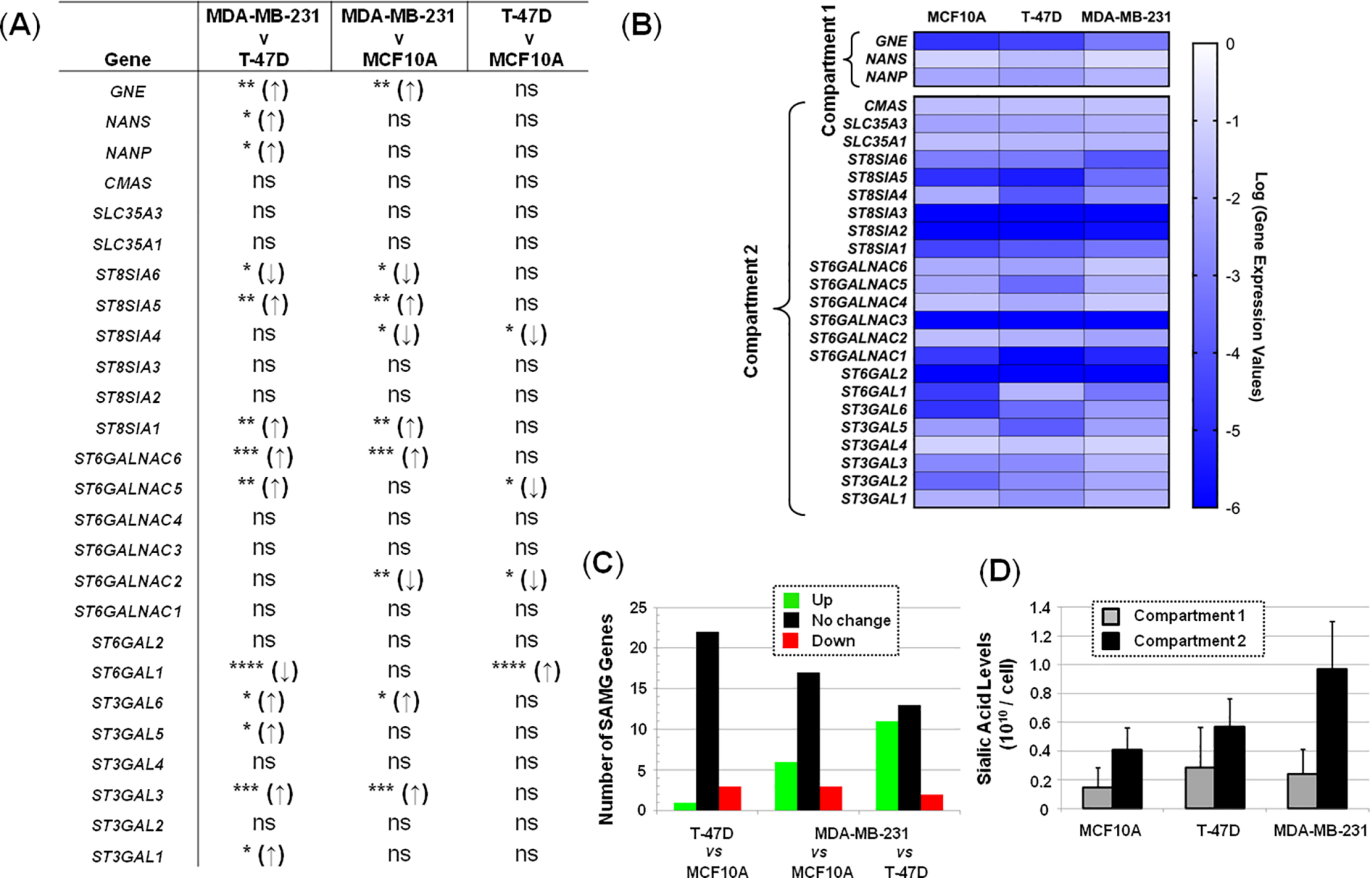
Comparison of SAMG mRNA levels in the MCF10A, T-47D, and MDA-MB-231 breast cell lines not treated with ManNAc analog. (**A**) Pair-wise statistical analysis, * p<0.05. (**B**) Heat map based on qRT-PCR analysis. (**C**) Number of genes up- or down-regulated (or not changed) in the given comparisons across cell lines. (**D**) Background sialic acid levels in each cell by ‘Compartment’ (as defined in Fig 1).

To provide a baseline for the subsequent evaluation of sialic acid production, we first investigated transcript levels of genes involved in (i) intracellular ‘Compartment 1’ sialic acid metabolism (*GNE*, *NANS*, *NANP*, and *CMAS*), (ii) transporters that translocate CMP-sialic acid (*SLC35A1* and *SLC35A3*) into the Golgi, and (iii) the 20 human sialyltransferases [24,72] in the absence of ManNAc analog supplementation (Fig 2). In this comparison the only difference found in ‘Compartment 1’ when comparing the MCF10A cells with either cancer line was associated with *GNE*, where this gene was more highly expressed in the MDA-MB-231 cells (Fig 2A). Interestingly, the three genes (*GNE*, *NANS*, and *NANP*) directly involved in intracellular sialic acid metabolism showed statistically-significantly differences when comparing the two cancer lines (T-47D and MDA-MB-231) with each other although the magnitude of the changes was modest (as indicated by the heat map shown in Fig 2B).

A larger number of statistically-significant ‘Compartment 2’ differences were observed when the MCF10A cells were compared with cancer lines including *ST3GAL3*, *ST3GAL6*, *ST6GAL1*, *ST6GALNAC2*, *ST6GALNAC5*, *ST6GALNAC6*, *ST8SIA1*, *ST8SIA4*, *ST8SIA5*, *ST8SIA6* (Fig 2A and 2B). Similarly, several sialyltransferases (*ST3GAL1*, *ST3GAL3*, *ST3GAL5*, *ST3GAL6*, *ST6GAL1*, *ST6GALNAC5*, *ST6GALNAC6*, *ST8SIA1*, *ST8SIA5*, and *ST8SIA6I)* showed differences when comparing the two cancer lines (i.e., T-47D *cf*. MDA-MB-231). The overall number of these genes differentially expressed when comparing the three cell lines is summarized in Fig 2C. The levels of sialic acid naturally found in cells (Fig 2D) are generally attributed to the combined action of these sets of enzymes and as a corollary, differences between cell types result from cell line-specific expression of the various SAMG genes.

### Transcript analysis of SAMG genes in analog-treated cells

**Fig 3:**
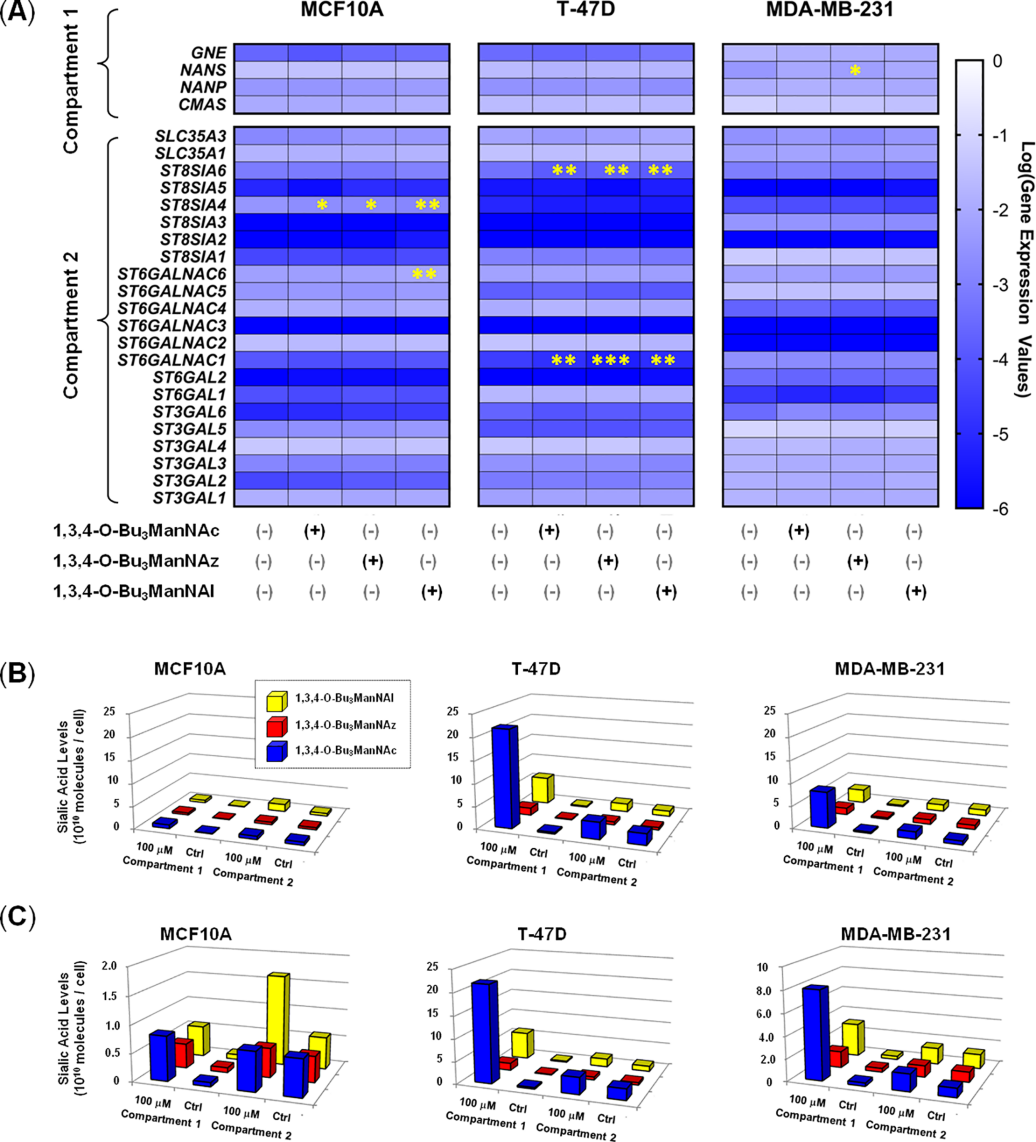
Transcript levels of SAMG genes and correlated sialic acid levels in ManNAc analog-treated cells. (**A**) Heat maps of SAMG genes associated with Compartment 1 and Compartment 2 in each of the breast cell lines upon analog treatment as indicated. (**B**) Compartment 1 and 2 levels of sialic acid in analog-treated cells (100 mM for 24 h) with the same data plotted in (**C**) with an expanded y-axis for the MCF10A and MDA-MB-231 lines. Although not readily apparent in the heat map color scheme, several minor but statistically-significant differences in transcript levels were observed as indicated by the asterisks (* p < 0.05, ** p < 0.01, *** p < 0.001 based on the statistical analysis presented in the supporting data **(Columns D-F in** S2 File).

Upon establishing baseline transcript levels in the panel of cell lines (Fig 2) we tested whether analog supplementation altered the expression of the SAMG genes, which could occur by two mechanisms. First, these compounds supply butyrate to a cell upon esterase processing [70,73]; this SCFA can act as an histone deacetylase (HDAC) inhibitor and alter gene expression epigentically [43]. Second, the transcript levels of sialyltransferases can be regulated by the bioavailability and biosynthesis of sialic acid [74], which is dramatically affected by analog supplementation [38,39]. We found, however, that the transcript levels of SAMG genes was only minimally affected by analog supplementation with only minor differences observed between control and analog-treated cells in the heat map representations (Fig 3).

### High-flux ManNAc analogs reveal differences in sialic acid biosynthesis

Upon verification that the ManNAc analogs showed minimal growth inhibition and negligible cytotoxicity (S2 Fig) and had minimal impact on SAMG transcript levels (Fig 3A), we measured sialic acid production upon analog supplementation using the periodate resorcinol assay. This method allows the total amount of sialic acid (Panel A in S3 Fig) to be compared with the free (i.e., unconjugated) form of this sugar (i.e., Compartment 1, Fig 1) as well as its subsequent incorporation into sialoglycoconjugates (Compartment 2). More specifically, Compartment 1 constitutes the unconjugated, free monosaccharide metabolites of sialic acid whose levels reflect the activities of CES, GNE, NANS, and NANP. Compartment 2 includes CMP-sialic acids produced by CMAS (which are typically present at negligible levels in ManNAc analog-treated cells compared to earlier pathway intermediates [48]) together with glycoconjugate-bound sialic acids produced by one or more of the 20 human sialyltransferases after CMP-sialic acid transport into the Golgi lumen by the SLC35A1 or SLC35A3 transporter. In the first set of experiments we used conditions (100 μM of analog evaluated after 24 h of exposure) that—based on previous testing across diverse cell lines [39,40,46]—that provide robust signal but do not saturate the sialic acid biosynthetic ability of the cells. In other words, the assays were conducted under conditions that provide near-linear analog concentration and time responses so that differences between analog utilization under the various conditions can be observed unambiguously. A comparison of sialic acid production where the y-axis was held constant across all three lines (Fig 3B) clearly shows that the MCF10A cell line has a very limited capacity to produce sialic acid compared to either of the two cancer lines. Another clear observation is that 1,3,4-O-Bu_3_ManNAc, which is the analog that produces the natural Neu5Ac form of sialic acid, supports much higher flux (especially in Compartment 1) than either of the non-natural analogs.

Additional insights into cell type-specific facets of sialic acid metabolism are apparent when the y-axis is adjusted to emphasize the full range of production in analog-treated cells (Fig 3C). For example, the two cancer lines (T-47D and MDA-MB-231) qualitatively exhibit similar metabolic characteristics (i.e., the *relative* ratios of each peak are roughly comparable). By contrast, several differences emerge that differentiate the MCF10A cells from the cancer lines; for example Compartment 1 production in 1,3,4-O-Bu_3_ManNAc-treated cells showed little increase whereas the novel non-natural 1,3,4-O-Bu_3_ManNAl analog almost tripled Compartment 2 levels of sialic acid. In addition to such cell line-specific features of analog utilization, certain trends held across all lines; for example in all cases the relative increase in sialic acid between untreated control cells and the analog-treated cells was much larger for Compartment 1 metabolites. More specifically, increases in Compartment 1 (free sialic acid) levels ranged from a *minimum* of ~5-fold for 1,3,4-O-Bu_3_ManNAz-treated MDA-MB-231 cells to > 50-fold for 1,3,4-O-Bu_3_ManNAc treated T-47D cells; by comparison the *largest* increase for Compartment 2 levels was ~3-fold (in the 1,3,4-O-Bu_3_ManNAl-treated MCF10A cells).

### Analysis of expanded conditions and determination of ‘Reserve Capacity’

**Fig 4:**
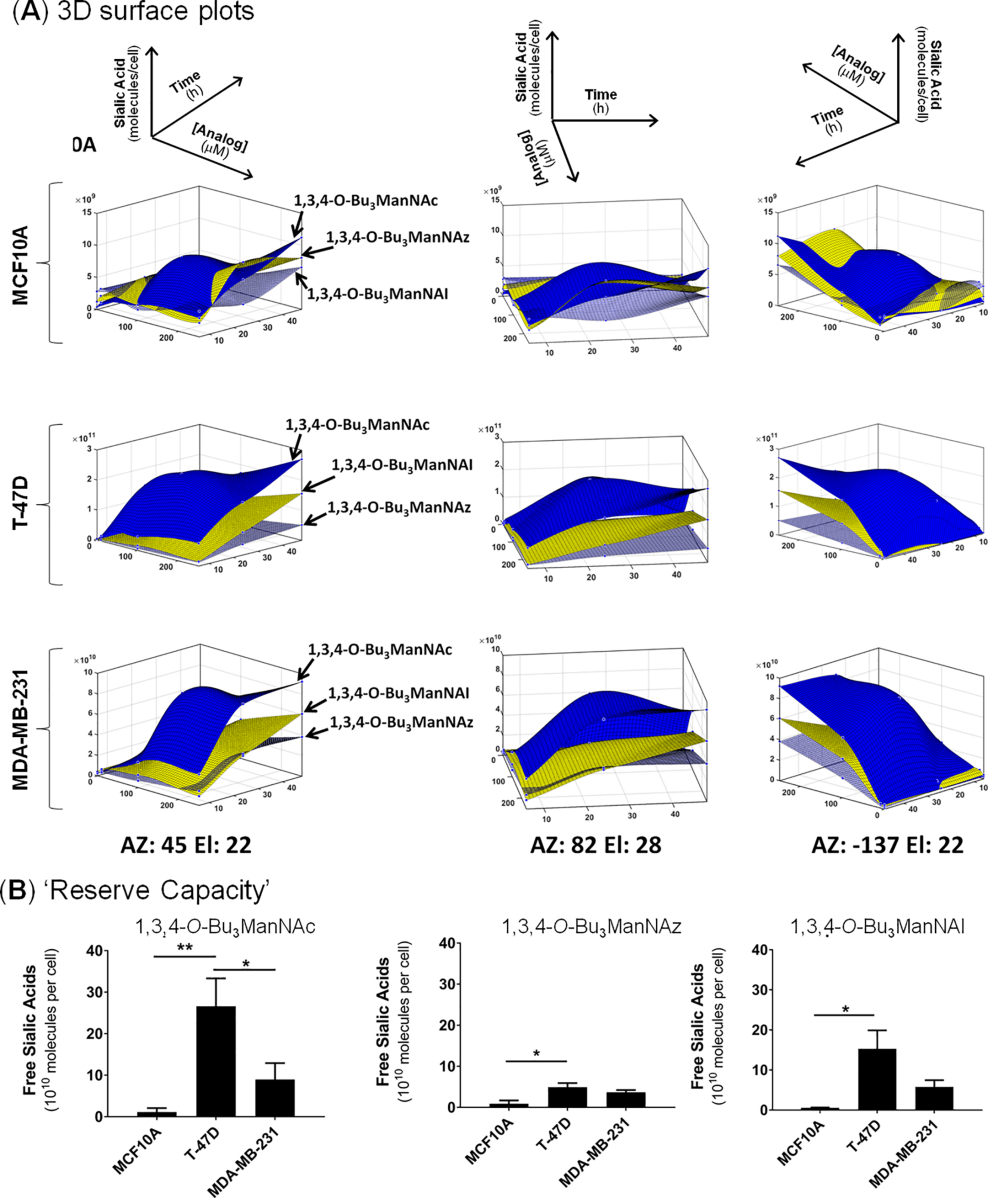
Metabolic profiles of Compartment 1 in analog-supplemented cells. (**A**) 3D surface plots were generated for each cell line in response to treatments with analogs; sialic acid production is shown as a function of both dose and time (AZ stands for azimuth values, El stands for elevation values, and the three plots provided for each data set are rotated to depict different vantage points). (**B**) The Reserve Capacity for each cell line was calculated based on the highest observed increase in Compartment 1 sialic acid levels in analog-supplemented cells compared to untreated controls.

After making the intriguing discovery that each cell line had a unique ability to produce sialic acid upon analog supplementation at a defined set of conditions (100 μM exposure for 24 h), we probed the limits of this ability by testing a range of analog concentrations (up to 250 μM) and incubation times (up to 48 h) to cover conditions previously shown to maximize sialic acid production [39,40,46]. In other words, we sought to determine the maximum amount of sialic acid each cell line could produce, which required the testing of a range of dose- and time- permutations. By using the high throughput periodate resorcinol assay, we generated multidimensional measurements of total, Compartment 1, and Compartment 2 levels of sialic acid in each cell line analog across time intervals and analog concentrations (S3 Fig). Again (i.e., as observed at 100 μM/24 h) the strongest response observed for sialic acid production was for Compartment 1; accordingly, much of our subsequent analysis focused on the relevant intracellular sialometabolites. For example, the complexity of intracellular sialic acid metabolism is illustrated by 3D surface plots that show the biosynthesis of this sugar as a combined function of analog type, dose, and time (Fig 4A).

The data provided by the periodate resorcinol assays was ‘mined’ to extract further insights into sialic acid biosynthesis. For example, the rate of sialic acid production over time was calculated from this data, revealing that for some cell:analog combinations production was fastest in the time interval between 0 and 6 h while for other combinations sialic acid was produced most rapidly between 6 and 24 h (S4 Fig; in most cases the rate of sialic acid production declined after 24 h, which is to be expected as the culture medium becomes analog-depleted). To simplify comparisons of the complex character of sialic acid metabolism in analog-treated cells (as depicted in the 3D surface plots Fig 4A), we calculated a parameter we call ‘Reserve Capacity,’ which is based on the largest increase in free sialic acid production over basal levels observed under any of the tested conditions (Fig 4B). In essence, this metric represents the otherwise hidden ability of cells to produce sialic acid when supplied with ManNAc analogs that bypass CMP-sialic acid feedback inhibition of GNE, the enzyme that normally supplies the sialic acid pathway with substrate by converting UDP-GlcNAc to ManNAc [75,76]. The Reserve Capacities of Compartment 1 were consistently higher in the T-47D and MDA-MB-231 lines (with the T-47D line exhibiting the highest value of any line), compared to the near-normal MCF10A, which had an order of magnitude smaller ability to convert analog into sialic acid species compared to either cancer line. Finally, the composition of the N-acyl group had an impact on Reserve Capacity with a consistent rank order of 1,3,4-O-Bu_3_ManNAc > 1,3,4-O-Bu_3_ManNAl > 1,3,4-O-Bu_3_ManNAz for sialic acid production.

### Compartment ratios

**Fig 5:**
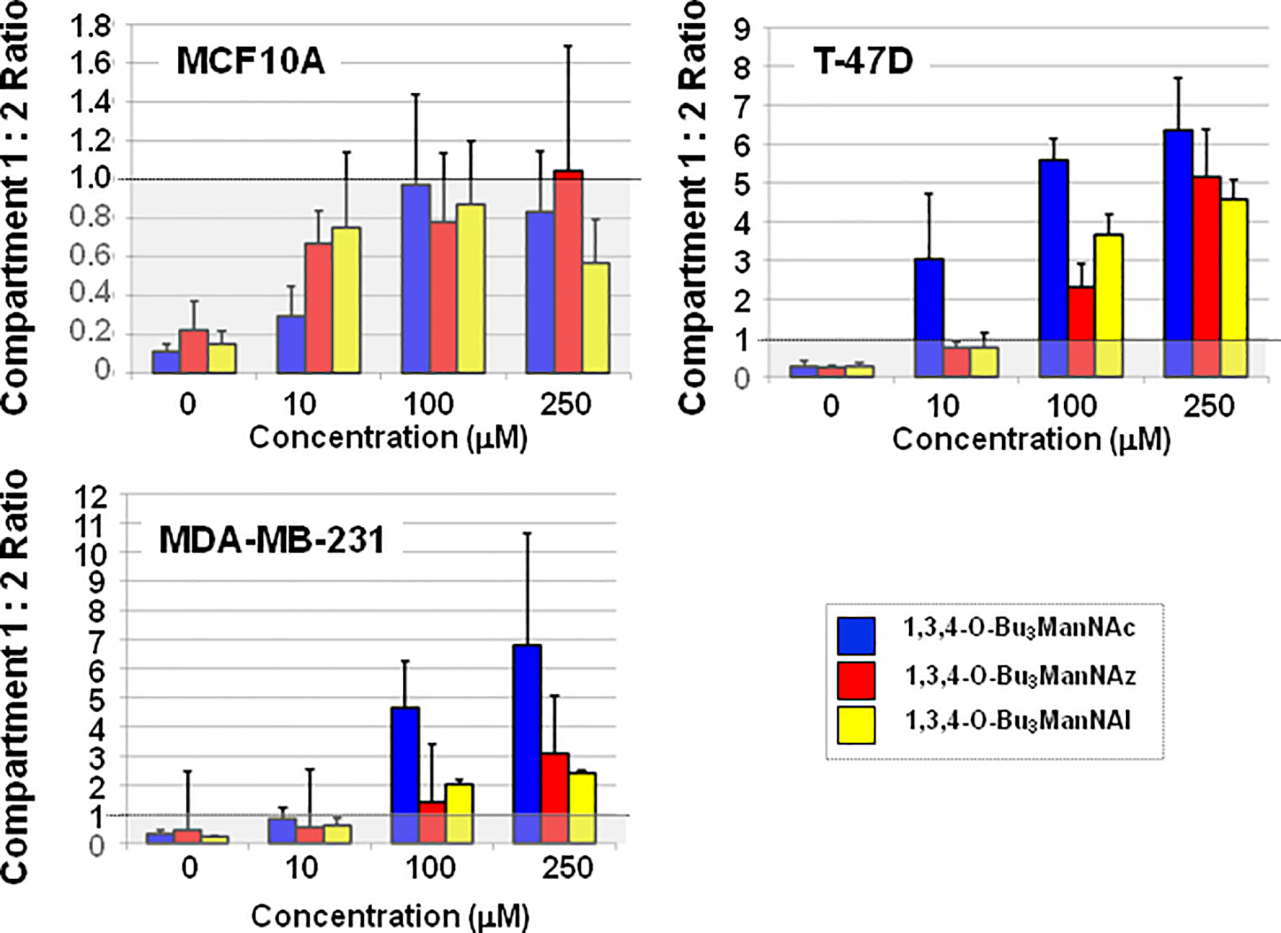
‘Compartment Ratios’ of ManNAc analog-treated cells. The ratio of sialic acid in Compartment 1 (i.e., unconjugated sialometabolites) compared to Compartment 2 (i.e., predominantly glycoconjugate-bound sialic acids) was calculated for cells after 24 h of treatment with each analog at the indicated concentrations.

Although changes in sialic acid levels in Compartment 2 were less pronounced than in Compartment 1 upon analog supplementation, this data provides useful information based on the ratios of sialic acid produced in each compartment (data for 24 h is shown in Fig 5; full data is given in supplemental S5 Fig). These ratios represent the respective biosynthetic abilities of each cell line to first covert ManNAc analogs into the corresponding sialic acid metabolites (i.e., Compartment 1 activity) and then to subsequently incorporate these sugars into sialoglycoconjugates (i.e., Compartment 2 activity). As expected, this ratio was less than one in all untreated (0 μM) cells, which reflects feedback control that restricts the amount of intracellular sialic acid made by cells when this sugar is not needed for sialoglycoconjugate production. In analog-supplemented cells, the Compartment 1 to Compartment 2 ratios consistently remained lower than one for the MCF10A line indicating that these cells do not have the capacity to produce large amounts of sialic acids even when the normal feedback mechanism (i.e., CMP-Neu5Ac inhibition of GNE [76]) is bypassed by feeding flux into the sialic acid pathway downstream of this regulatory enzyme. In other words, these near normal cells appear to have regulatory controls beyond feedback inhibition of GNE that strictly limit sialic acid production to levels commensurate with the cells’ requirements for sialoglycoconjugate production. By contrast the T-47D and MDA-MB-231 lines had Compartment 1 to Compartment 2 ratios that typically exceeded one, often by several fold, indicating that these cancer lines were able to covert exogenously-supplied ManNAc into sialic acid at levels that far the amount of this sugar required for subsequent sialoglyconjugate production.

### Incorporation of analog-derived flux into sialoglycoconjugates

**Fig 6:**
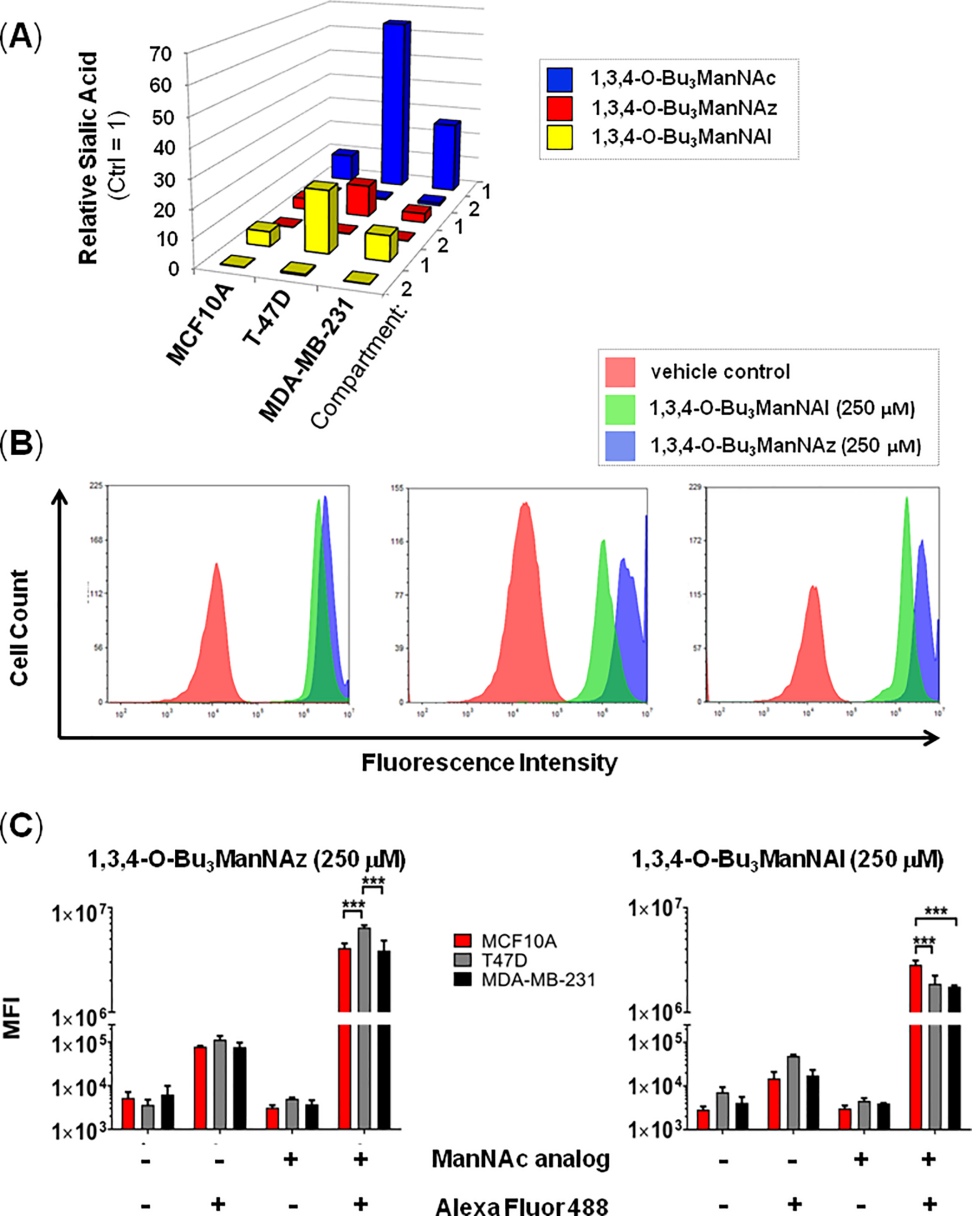
Verification of metabolic incorporation of sialometabolites into Compartment 2 glycoconjugates. (**A**) Relative levels of sialic acid species in each cell line in Compartment 1 and 2 after analog treatment (250 mM for 24 h) compared to untreated control cells are summarized to show the comparatively minor perturbation of Compartment 2 in all cases. (**B**) The presence of azido- or alkyne-modified sialic acids on the surfaces of cells was determined by labeling cells with the appropriate complementary fluorescent probe followed by FACS to quantify the overall abundance of each type of non-natural sialic acid after incorporation into sialoglyconjgates (i.e., O-linked or N-linked glycoproteins or gangliosides, as shown in Fig 1). The flow cytometry results showed very low background signal unless both the analog and labeling agent were present, indicating that robust incorporation of sialosides into sialoglycoconjugates did occur. *** indicates p < .001, error bars represent ± SEM.

In a final set of experiments, we used flow cytometry to verify incorporation of the exogenously-supplied analogs into Compartment 2 components (i.e., sialoglycoconjugates). The motivation for these experiments were twofold; first ManNAc can increase GNE activity [77,78], establishing a formal (although unlikely) possibility that the high levels of sialic acid metabolites observed in analog-treated cells was a consequence of increased conversion of UDP-GlcNAc to ManNAc by this enzyme. Second, the highly-elevated levels of Compartment 1 metabolites (where several 10-fold or higher changes were observed) were not reflected in Compartment 2 where almost all increases were by 0.5-fold or less (as illustrated in Fig 6A). Biologically, this response is plausible provided that metabolite from the exogenous analog largely replaces, rather than augments, endogenous metabolic flux into Compartment 2. Unambiguous verification that such analog incorporation did occur for 1,3,4-O-Bu_3_ManNAz and 1,3,4-O-Bu_3_ManNAl was obtained by bioorthogonal ‘click’ conjugation reactions that only label glycoconjugates derived from exogenously supplied non-natural analogs (representative histograms are shown in Fig 6B with quantification of multiple experiments provided in Fig 6C).

In all three cell lines, 1,3,4-O-Bu_3_ManNAz supplementation resulted in cell surface labeling at least 2-fold higher than observed in the 1,3,4-O-Bu_3_ManNAl-treated cells; interestingly this ‘Compartment 2’ result showed the opposite trends as the ‘Compartment 1’ data given earlier (S3 Fig) where the biosynthesis of intracellular sialic acid metabolites was higher in the 1,3,4-O-Bu_3_ManNAl supplemented cells than in cells incubated with 1,3,4-O-Bu_3_ManNAz. Furthermore, the surface display of non-natural sialic acids was cell line specific and depended on whether the azido or alkyne analog was used (e.g., T-47D cells had stronger labeling with 1,3,4-O-Bu_3_ManNAz whereas the MCF10A cells had more robust labeling with 1,3,4-O-Bu_3_ManAl (Fig 6C). Interestingly, despite the increased production of intracellular alkyne-modified sialometabolites in 1,3,4-O-Bu_3_ManNAl-treated cells compared to 1,3,4-O-Bu_3_ManNAz-treated cells (e.g., as shown in Fig 3C and 3D), the Compartment 2 biosynthetic machinery more efficiently incorporated the lower levels of azide-modified Compartment 1 metabolites produced in 1,3,4-O-Bu_3_ManNAz-treated cells into the Compartment 2 sialoglycoconjugates detected in the flow cytometry assays. Of note, we ruled out one potential explanation for these analog-specific observations, which was altered transcription of the repertoire of Compartment 2 SAMG genes upon supplementation (Fig 3A). A biochemical explanation for this observation may lie in enhanced substrate promiscuity for Golgi CMP-Neu5Ac transporters or sialyltransferases that allows preferential incorporation of CMP-Sia5Az into sialoglycans.

### Distinction between cell subtypes based on the integration of genetic and metabolic data

The distinctive analog-, dose-, and time-specific metabolic features exhibited by each cell line (as documented in Figs 2–6) suggested that our MGE-based strategy had the potential to distinguish cell type provided that the differences could be quantified appropriately. To accomplish this task we developed a multiple regression model to predict cell type that involved both inherent genetic features of the three cell lines (i.e., transcription of *CMAS*, *SLC35A1*, *SLC3A3*, and *NANP* because they had the most significant differences in the absence of analog supplementation) and metabolic features illuminated by analog supplementation (i.e., Compartment 1 Reserve Capacity for each of the three analogs in each of the three cell lines, which is a parameter that summarizes analog metabolism). These features were selected not only because they covered by genetic and metabolic attributes of the cells, but because they exhibited the most striking differences in the large number of potential features available to be modeled. The regression model was developed for gene expression only, metabolite features only, as well as both combined. Using these features, we trained the multiple regression model and determined intercept and feature coefficient values (Table 1).

**Table 1:** Linear regression model with calculated parameters.

Feature	Gene Feature Coefficients	Metabolite Feature Coefficients	Combined Coefficient
Intercept	2.58	7.20e-1	1.79
*CMAS*	20.10	N/A	-4.24e+1
*SLC35A1*	-80.41	N/A	-3.47e+1
*SLC35A3*	-9.97	N/A	1.88e+1
*NANP*	62.00	N/A	1.27e+2
Compartment 1 Reserve Capacity (1,3,4-O-Bu_3_ManNAc)	N/A	1.825e-12	2.40e-12
Compartment 1 Reserve Capacity (1,3,4-O-Bu_3_ManNAz)	N/A	5.75e-11	9.51e-12
Compartment 1 Reserve Capacity (1,3,4-O-Bu_3_ManNAl)	N/A	-1.33e-11	2.09e-13

We implemented the model in three ways; the first was based only on the gene features (i.e., the expression of CMAS, SLC35A1, SLC35A3, and NANP) in cells not treated with analog. When using only these features to conduct F-tests designed to assess overall significance we obtained *p* = 0.21. Similarly, implementation of the model based only on the metabolite features yielded a non-significantly significant result (*p* = 0.15). By contrast the model returned a highly significant result (*p* = 0.0039) based on a combination of both the genetic and metabolite features.

## Discussion

Our group has a longstanding interest in the contributions of sialic acid metabolism to cancer. For example, in previous MGE-based glycoproteomics studies we demonstrated that supplementation of pancreatic cancer cells with 1,3,4-O-Bu_3_ManNAc or 1,3,4-O-Bu_3_ManNAz can selectively target subsets of glycoproteins for increased [47] or non-natural sialylation [79]. We have also explored the role of sialylation in breast cancer [43,44,57,58], helping to further explain how abnormalities in this sugar’s metabolism and glycoconjugate display contribute to this disease [14,80,81]. Based on the consolidating evidence that metabolic flux contributes to cancer progression, in this study we demonstrated that the ‘high-flux’ ManNAc analogs our team has developed have the potential for uncovering otherwise hidden features of sialic acid metabolism. We specifically focused on breast cancer by integrating transcription of sialic acid metabolism and glycosylation (SAMG) genes with the impact of altered metabolic flux in near-normal breast cells (MCF10A) compared with increasingly malignant cells (the T-47D and MDA-MB-231 cancer lines [82]) using ‘high flux’ ManNAc derivatives including the previously unreported alkyne-modified analog 1,3,4-O-Bu_3_ManNAl.

Evaluation of SAMG gene transcription across the three cell lines (Fig 2A and 2B) in the absence of the metabolism-modifying analogs found differences that support the generally accepted premise that sialic acid is linked to advanced stage cancer. For example, a comparison of the earlier stage T-47D cancer cells with the near normal MCF10A line showed that out of the 26 SAMG genes, only four were differently expressed in the cancer cells (Fig 2C; one was up-regulated and three were down-regulated). By comparison, a larger number SAMG genes were differentially expressed in the MDA-MB-231 line compared with either the MCF10A or T-47D lines with the trend being towards increased expression, especially of STs. These observations are consistent with the higher level of glycoconjugate-bound sialic acid found in the advanced stage MDA-MB-231 cancer cells in this study (Compartment 2, Fig 2D) as well as the many contributions of sialic acid to cancer progression reported in previous studies (as discussed earlier in the Introduction).

Specific differences in SAMG gene expression between the three cell lines relevant to cancer include sialyltransferases linked to poor outcomes in breast cancer. For example, *ST6GAL1* is a proto-oncogene linked to poor prognosis across an array of cancers (including breast) where expression is correlated with promoter methylation [83]. In the current study, this gene—which regulates stem cell transcription factors and confers a cancer stem cell phenotype—was highly over-expressed in the T-47D cells and then down-regulated in the more advanced MDA-MB-231 line. Similarly, differences in transcription of the *ST6GALNAC* family of STs were observed between the MCF10A and the cancer lines T-47D and MDA-MB-231 as well as between the cancer lines themselves (i.e. T-47D versus MDA-MB-231); such genetic alterations have been linked to the metastasis of breast cancer to the brain [84,85]. Additionally, *ST3GAL1* can be hypomethylated leading to transcriptional activation in invasive breast ductal carcinoma [83]; this gene was overexpressed in MDA-MB-231 cells in the current study.

A second facet of the transcriptional profiling of the breast cells was that treatment with 1,3,4-O-Bu_3_ManNAc, 1,3,4-O-Bu_3_ManNAz, or 1,3,4-O-Bu_3_ManNAl had minimal impact on SAMG gene expression with only one or two genes affected in any of the cell lines (Fig 3). This result is roughly consistent with the impact of sialic acid flux (modulated by GNE knock out) in embryonic murine cells where a minority (3 or 4) of all STs were affected [74] and a previous genome-wide transcriptional analysis of ‘1,3,4’-tributanoyl-modified hexosamines in breast cancer cells where only minor changes in transcription were observed [45]. The finding that SAMG gene transcription is only minimally perturbed upon analog supplementation indicates that changes in sialometabolite abundance (i.e., ‘Compartment 1’ components) or sialoglycoconjugate production (‘Compartment 2’) primarily result from flux-based mechanisms in the treated cells rather than genetic control. An example of the disconnect between metabolic flux and genetic control is illustrated by intracellular sialometabolite levels, which were dramatically higher in the T-47D line, and strongly higher in the MDA-MB-231 line, compared to the near normal MCF10A cells upon analog supplementation. These results—which are readily visualized by comparing either the ‘Reserve Capacity’ (Fig 4) or the ratios of sialic acid present in Compartment 1 and 2 after analog treatment (Fig 5A)—are noteworthy because the capacity for sialic acid production does not correspond to the relative expression levels of the intracellular pathway genes. In particular, these ‘Compartment 1’ genes were expressed at comparable levels in the MCF10A cells and the cancer lines either with (Fig 3) or without (Fig 2) analog supplementation, with only the difference being slightly higher *GNE* transcription in the MCF10A cells. Interestingly, this difference in *GNE* where lower expression was observed in the T-47D line, ran counter to the observed metabolite levels where much higher levels of sialic acid were produced compared to MCF10A cells. The response in the MDA-MB-231 cells was even more curious with *GNE*, *NANS*, and *NANP* all upregulated compared to the T-47D cells, yet the MDA-MB-231 cells had less than half the Compartment 1 ‘Reserve Capacity’ (i.e., latent biosynthetic ability) when supplemented with either 1,3,4-O-Bu_3_ManNAc or 1,3,4-O-Bu_3_ManNAl (Fig 5). These results implicate an as-of-yet unknown controlling mechanism that limits the production of sialometabolites not only in normal cells (i.e., in the MCF10A line compared to either cancer line) but also in certain cancer cells (i.e., in the MDA-MB-231 line compared to T-47D cells). Alternatively, the T-47D line may exploit an unknown mechanism that enhances sialic acid production; for example, to speculate, the activity of one or more pathway enzymes may be under post-translational control in a way that responds to sugar metabolism (a conceptual example is provided by c-Myc where O-GlcNAc modification prolongs the half-life of this oncoprotein with a commensurate increase in activity [86]).

The Compartment Ratio and Reserve Capacity data, which are metrics we devised to help make sense of the complex metabolic features of analog processing across the three test cell lines, are consistent with certain roles known for sialylation in cancer progression but also diverge in some respects. For example, the reluctance of MCF10A cells to convert the ‘high flux’ ManNAc analogs into copious amounts of sialic acid is consistent with the idea that normal cells do not require large amounts of this proto-oncogenic metabolite. By contrast, an unexpected finding was the ability of T-47D cells to produce larger amounts of sialic acid than the more advanced stage MDA-MB-231 line because cancer progression is generally associated with increasing sialylation and therefore logically the most highly advanced cancer cells (in this study, the MDA-MB-231 line) would be expected to have the highest capacity to produce this sugar. A speculative hypothesis is that earlier-stage cancer cells require a high capacity for sialic acid production to drive oncogenesis that, upon being achieved, is no longer needed and is thus attenuated in more advanced stage cancer. It is even possible that advanced stage cancer cells develop protective mechanisms to prevent hypersialylation, which has counter-intuitively been shown to hinder cancer cell viability in some circumstances. One example where hypersialylation can be counterproductive in cancer, our group has shown that sialic acid derived from 1,3,4-O-Bu_3_ManNAc sensitizes drug-resistant pancreatic cancer cells to tyrosine kinase inhibitors (erlotinib and gefitinib [22]) through attenuation of EGFR signaling [23].

The ability of the ‘high flux’ ManNAc analogs to illuminate otherwise hidden metabolic features of human breast cell subtypes based on the slight chemical changes present at their N-acyl groups (e.g., ‘NAc’ vs ‘NAz’ vs ‘NAl’; Fig 1) raises the possibility that this class of compounds could be exploited for the detection or diagnosis of cancer. (The need for improved diagnostic approaches for breast cancer is based in part on the current over-diagnosis and over-treatment of this disease [87,88]). By using metabolic metrics including the capacity for sialic acid biosynthesis, which we showed can discriminate cell lines representing different stages of cancer progression using a multiple regression model, we propose that diagnostic precision can be improved if metabolic characteristics related to sialic acid biosynthesis explored in this report hold broadly across normal, earlier, and later stage cancer. To emphasize, to gain clinical relevance, the findings given here characterizing the metabolic and genetic profiles of near normal, earlier, and advanced cancer lines will need to be validated across many additional lines representing each stage cancer and it is also likely that the ‘features’ selected for regression modeling to discriminate between the lines will need to be fine tuned as additional data is acquired.

In summary, we have performed proof-of-principle experiments comprising a comparative analysis of sialic acid metabolism driven by supplementation of ‘high-flux’ ManNAc analogs across a panel of breast cells representing the transition from normal-to-highly metastatic breast cancer. This approach uncovered new molecular features of cancer, such as the hidden ‘Reserve Capacity’ of the sialic acid biosynthetic pathway for producing sialic acid, that when integrated with other types of datasets, such as gene expression profiling, provides a new method to discriminate between cell subtypes that ultimately may improve the treatment and diagnosis of cancer.

## Supporting information

S1 Fig Synthesis scheme and characterization of 1,3,4-O-Bu_3_ManNAl. (DOCX)

S2 Fig Growth inhibition and cytotoxicity. Each analog (1,3,4-O-Bu_3_ManNAc (**A**), 1,3,4-O-Bu_3_ManNAz (**B**), 1,3,4-O-Bu_3_ManNAl (**C**)) was screened for overt cytoxicity by monitoring growth rates by incubating MCF10A, T-47D, and MDA-MB-231 cells with 0, 10, 100, and 250 μM concentrations of each analog and evaluating cell counts at 6, 24, and 48 h. None of the three analogs had a statistically measurable impact on cell growth compared to controls. Error bars represent ± SEM.
(DOCX)

S3 Fig Determination of sialic acid levels in analog-treated cells using the periodate resorcinol assay. Each cell line (MCF10A, (i); T-47D (ii), and (MDA-MB-231 (iii)) was incubated with each analog (1,3,4-O-Bu_3_ManNAc, 1,3,4-O-Bu_3_ManNAz, or 1,3,4-O-Bu_3_ManNAl) at concentrations of 0, 10, 100, and 250 μM concentrations for 6, 24, and 48 h. The total levels of sialic acid are shown in Panel A (this page), the free monosaccharide (i.e., Compartment 1) levels in Panel B (Page 6), and the glycoconjugate bound (i.e., Compartment 2) levels in Panel C (Page 7).
(DOCX)

S4 Fig Calculation of the rate of sialic acid production in early (0 to 6 h), mid (6 to 24 h), and extended (24 to 48 h) time intervals after analog supplementation. The change in the number of sialic acid molecules per cell per minute was calculated for each cell line (MCF10A, T-47D, and MDA-MB-231) for each cell line for the indicated time intervals after addition of 0, 10, 100, or 250 μM of each analog (1,3,4-O-Bu_3_ManNAc, 1,3,4-O-Bu_3_ManNAz, or 1,3,4-O-Bu_3_ManNAl) at time = 0 h. The rates of production (with negative values indicating a decrease in sialic acid during the indicated time interval) are shown in Panel A (this page) for 1,3,4-O-Bu_3_ManNAc, in Panel B (Page 9) for 1,3,4-O-Bu_3_ManNAz, and in Panel C for 1,3,4-O-Bu_3_ManNAl (Page 10).
(DOCX)

S5 Fig Ratios of sialic acid production in Compartment 1 to Compartment 2 in ManNAc analog-supplemented cells. (DOCX)

S1 File Regression model input. (XLSX)

S2 File Gene expression statistical analysis. (XLSX)

S1 Table List of validated primers for qRT-PCR analysis of SAMG genes. (DOCX)
