# Supplementary material for: Integration of genetic and metabolic features related to sialic acid metabolism distinguishes human breast cell subtypes
Source: PLoS One. 2018 May 30;13(5):e0195812. doi: 10.1371/journal.pone.0195812 (PMC5976204; doi:10.1371/journal.pone.0195812)
Supplement: S1 Fig — (DOCX) [file pone.0195812.s001.docx]

**Synthesis and characterization of 1,3,4-O-Bu_3_ManNAl**


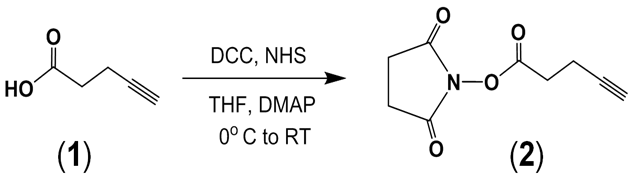


Compound (**1**) (500 mg) was mixed with 1.15 g of DCC (1.1 eq) and 645 mg of NHS (1.1 eq) and stirred in dry THF. A catalytic amount of DMAP was added and the reaction was stirred for 72 h at 0^0^C. The reaction was filtered, concentrated and purified with 50:50 ethyl acetate:hexane. White powder (742 mg) was obtained (75% yield). ^1^H NMR (500 MHz, chloroform-d) δ 2.76 - 2.99 (m, 6H), 2.53 - 2.71 (m, 2H), 2.06 (t, *J* = 2.75 Hz, 1H); ^13^C NMR (126 MHz, chloroform-d) δ 168.9, 167.0, 80.9, 70.1, 30.4, 25.6, 14.2.

**
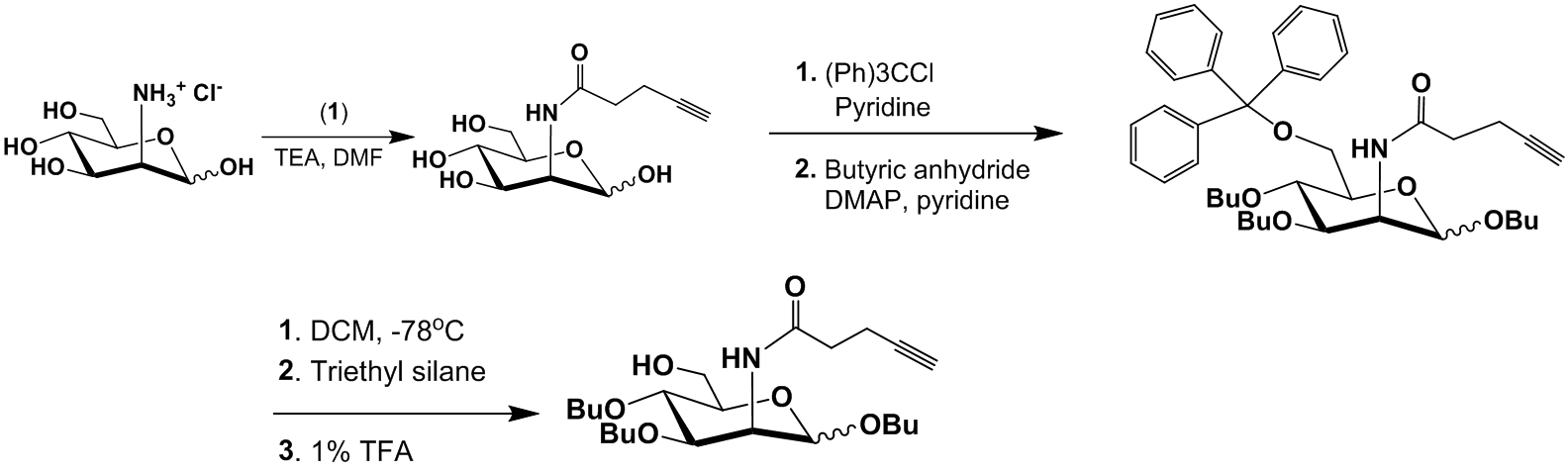
**

Mannosamine hydrochloride (860 mg) was stirred in 30 mL of DMF with 1.67 mL triethylamine (TEA) for 1 h. Compound (**2**) (742 mg) was added and the reaction was stirred over night. The reaction was concentrated, and the crude material was dissolved in 20 mL of pyridine and 1.0 g of trityl chloride was added. The reaction was stirred for 24 h and purified with flash chromatography using hexanes and ethyl acetate. The product was stirred in 20 mL of pyridine and butyric anhydride (6.0 eq.) was added followed by a catalytic amount of DMAP. The reaction was stirred overnight, concentrated, and purified on a flash column using ethyl acetate:hexanes. Deprotection of the trityl group was performed by dissolving the tributanoylated protected product (800 mg) in dicholormethane (20 mL) at -78^0^C. Triethyl silane (1.0 mL) was added dropwise (1.1 eq) while stirring followed by 1.5 mL of 1% TFA. The reaction was stirred for 30 min after which time the product was extracted with ethyl acetate and saturated sodium bicarbonate followed by flash chromatography purification using ethyl acetate:hexanes. Product (1,3,4-O-Bu_3_ManNAl was obtained (1.13 g, 47.7% yield). ^1^H NMR (500 MHz, chloroform-d) δ 6.07 (d, *J* = 8.49 Hz, 1H), 5.49 (t, *J* = 3.62 Hz, 1H), 5.14 (dd, *J* = 3.38, 8.57 Hz, 1H), 5.00 (dd, *J* = 1.57, 3.93 Hz, 2H), 4.36 (dt, *J* = 1.49, 6.64 Hz, 2H), 4.15 (dd, *J* = 1.65, 6.52 Hz, 2H), 2.15 - 2.49 (m, 17H), 1.42 - 1.83 (m, 18H), 0.82 - 1.07 (m, 25H); ^13^C NMR (126 MHz, chloroform-d) δ 173.1, 172.1, 172.0, 171.8, 171.6, 169.5, 89.9, 71.5, 67.4, 67.3, 67.3, 61.3, 37.2, 36.0, 36.0, 35.9, 35.8, 35.4, 18.5, 18.4, 18.3, 18.1, 17.8, 13.6, 13.6, 13.5, 13.5.
