# Supplementary figures and images for: Integration of genetic and metabolic features related to sialic acid metabolism distinguishes human breast cell subtypes
Source: PLoS One. 2018 May 30;13(5):e0195812. doi: 10.1371/journal.pone.0195812 (PMC5976204; doi:10.1371/journal.pone.0195812)

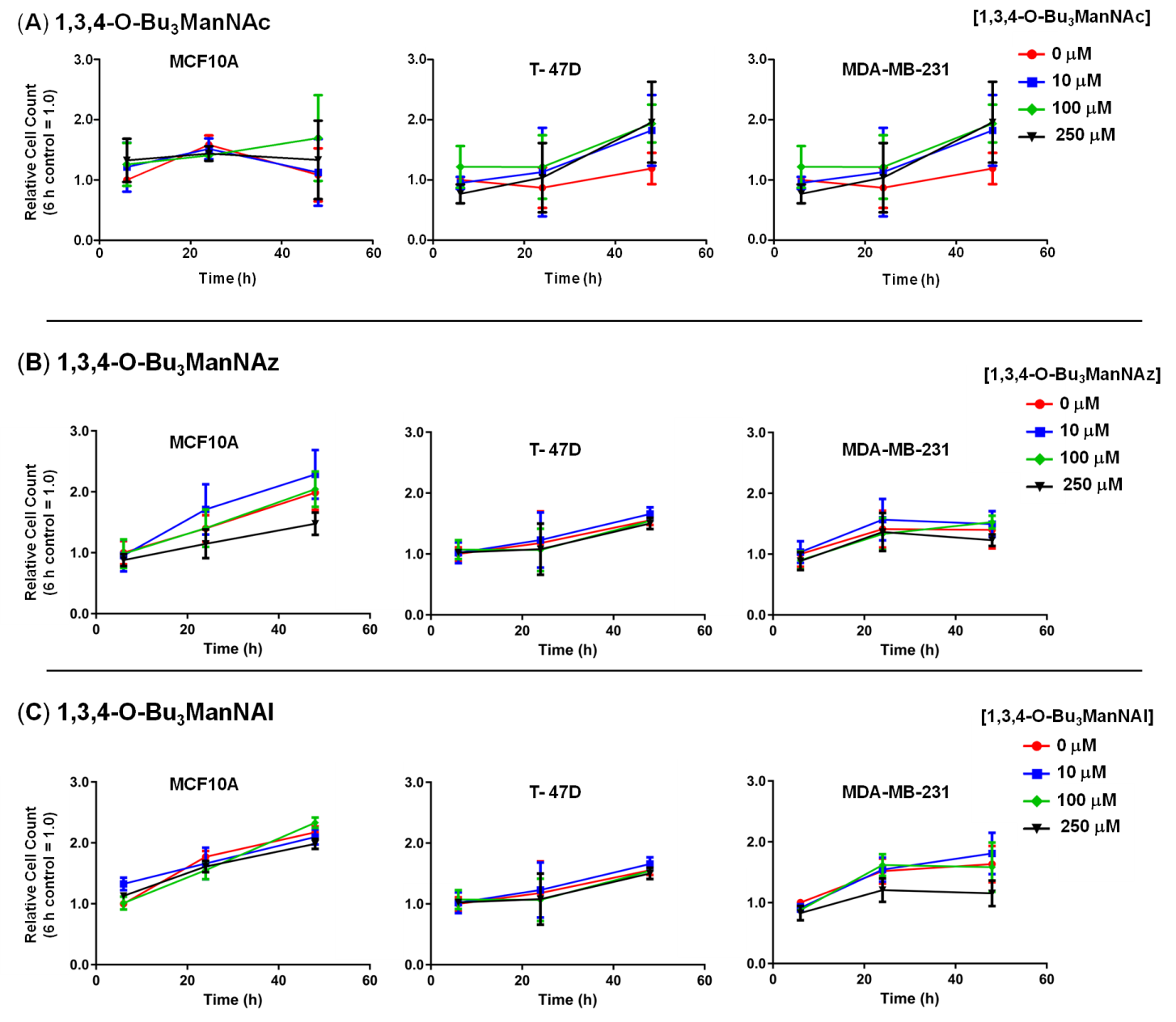

Supplement: S2 Fig — Each analog (1,3,4-O-Bu3ManNAc (A), 1,3,4-O-Bu3ManNAz (B), 1,3,4-O-Bu3ManNAl (C)) was screened for overt cytoxicity by monitoring growth rates by incubating MCF10A, T-47D, and MDA-MB-231 cells with 0, 10, 100, and 250 μM concentrations of each analog and evaluating cell counts at 6, 24, and 48 h. None of the three analogs had a statistically measurable impact on cell growth compared to controls. Error bars represent ± SEM. (DOCX) [file pone.0195812.s002.docx]

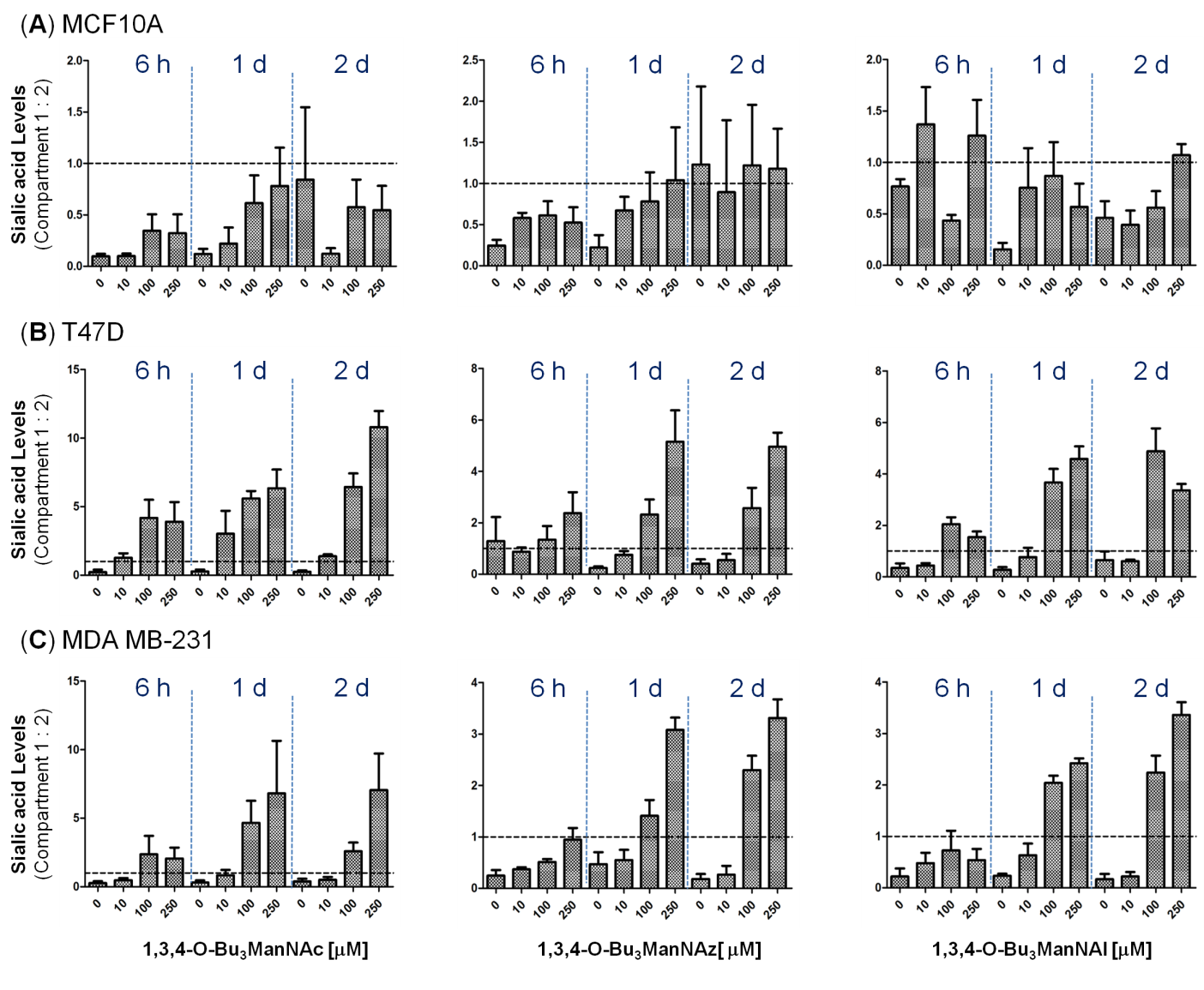

Supplement: S5 Fig — (DOCX) [file pone.0195812.s005.docx]

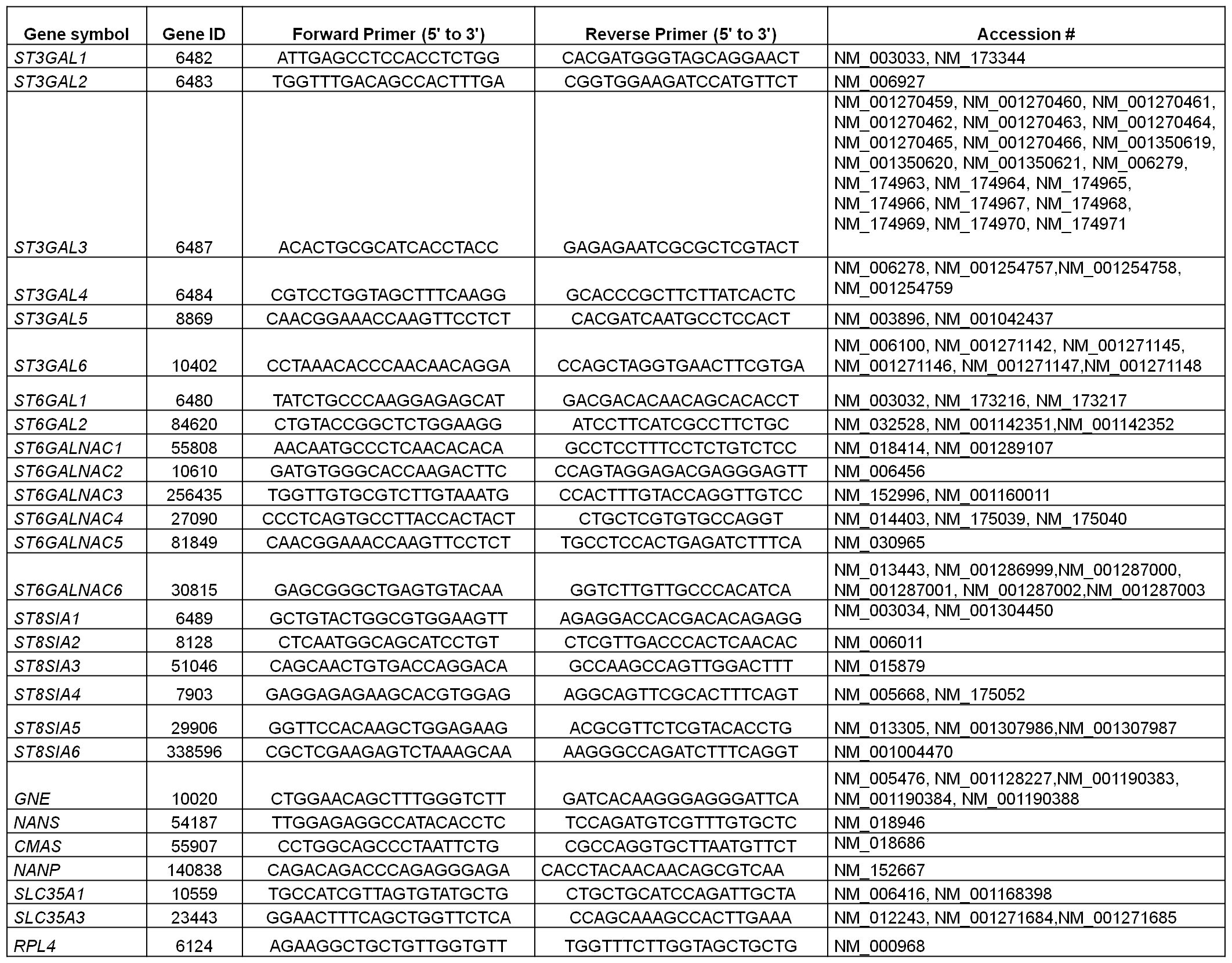

Supplement: S1 Table — (DOCX) [file pone.0195812.s008.docx]
