# Supplementary material for: Integration of genetic and metabolic features related to sialic acid metabolism distinguishes human breast cell subtypes
Source: PLoS One. 2018 May 30;13(5):e0195812. doi: 10.1371/journal.pone.0195812 (PMC5976204; doi:10.1371/journal.pone.0195812)

**(A) Total sialic acid in ManNAc analog-treated breast cell lines**:


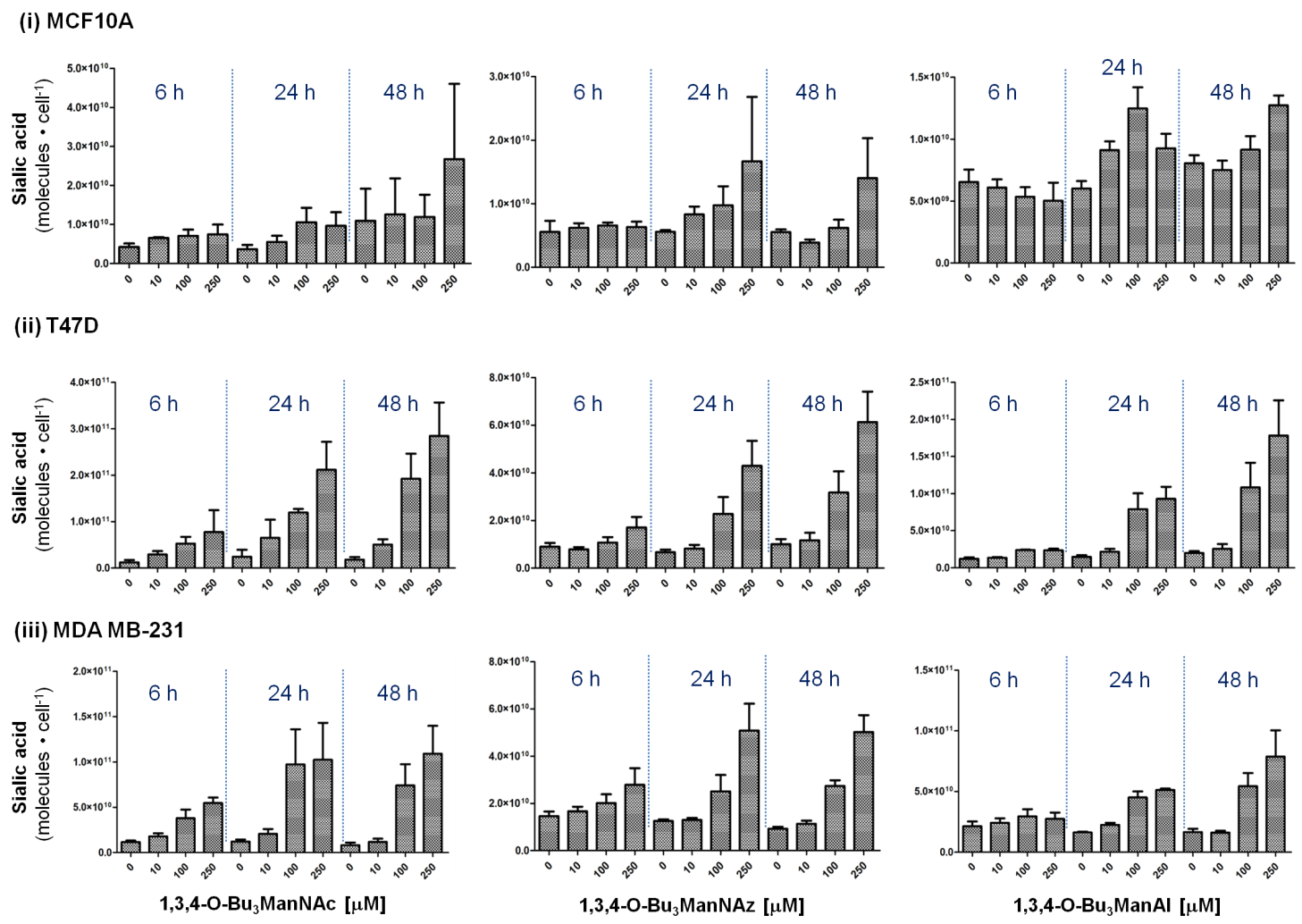


**(B) Free monosaccharide sialic acid in ManNAc analog-treated breast cell lines**:


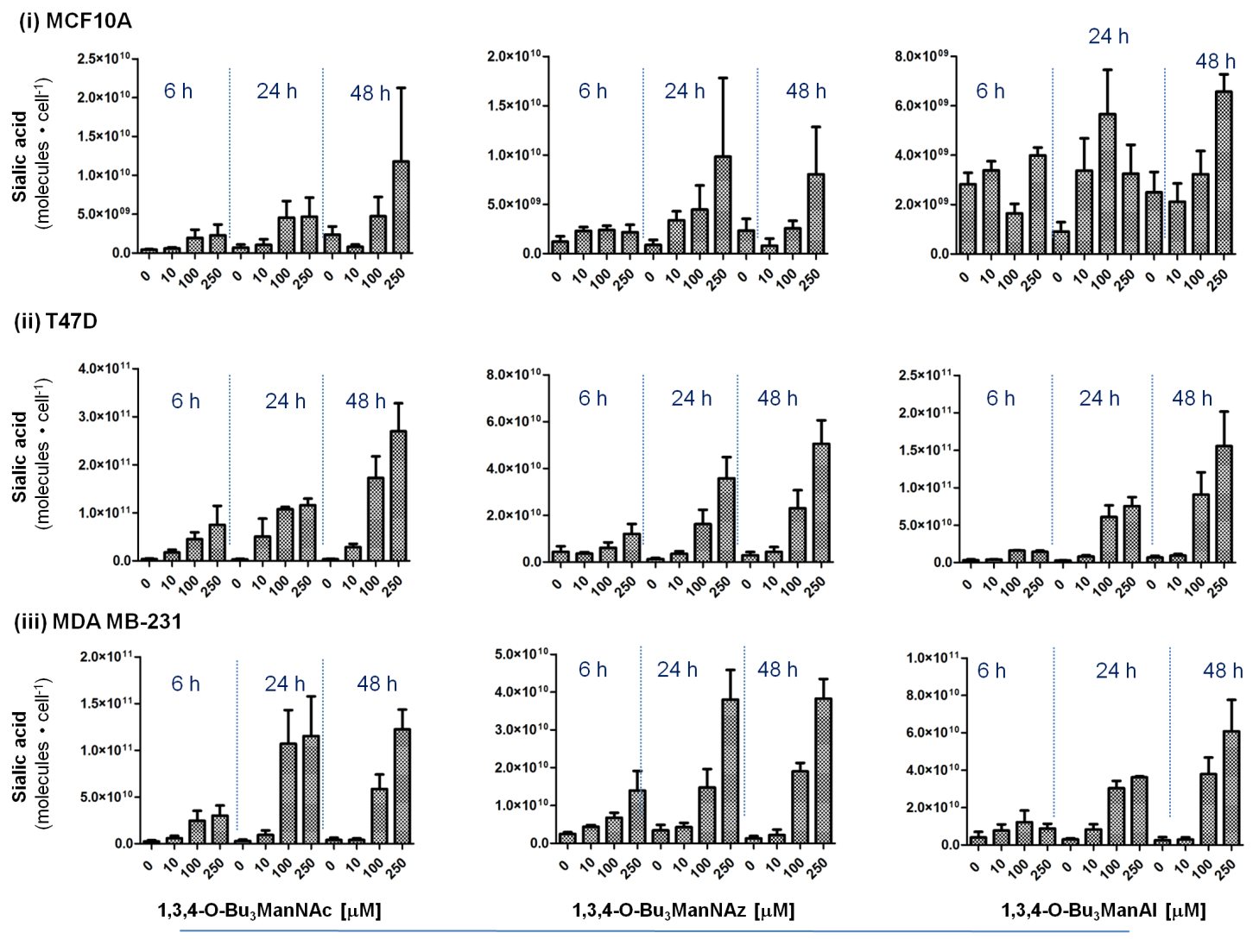


**(C) Glycoconjugate bound sialic acid in ManNAc analog-treated breast cell lines**:


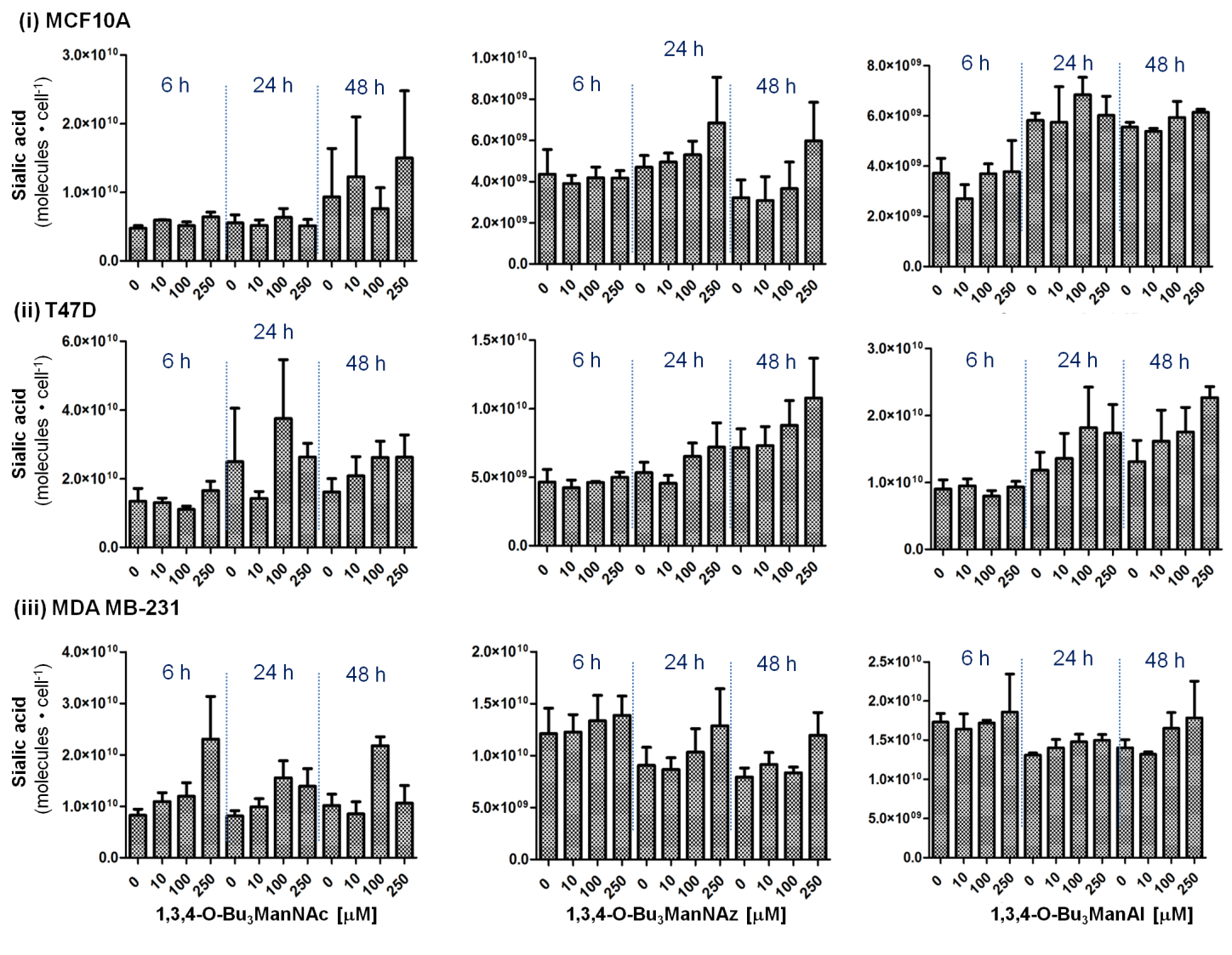

Supplement: S3 Fig — Each cell line (MCF10A, (i); T-47D (ii), and (MDA-MB-231 (iii)) was incubated with each analog (1,3,4-O-Bu3ManNAc, 1,3,4-O-Bu3ManNAz, or 1,3,4-O-Bu3ManNAl) at concentrations of 0, 10, 100, and 250 μM concentrations for 6, 24, and 48 h. The total levels of sialic acid are shown in Panel A (this page), the free monosaccharide (i.e., Compartment 1) levels in Panel B (Page 6), and the glycoconjugate bound (i.e., Compartment 2) levels in Panel C (Page 7). (DOCX) [file pone.0195812.s003.docx]
