# Supplementary material for: Integration of genetic and metabolic features related to sialic acid metabolism distinguishes human breast cell subtypes
Source: PLoS One. 2018 May 30;13(5):e0195812. doi: 10.1371/journal.pone.0195812 (PMC5976204; doi:10.1371/journal.pone.0195812)

**(A) Rate of sialic acid production in 1,3,4-O-Bu_3_ManNAc-supplemented breast cell lines**:


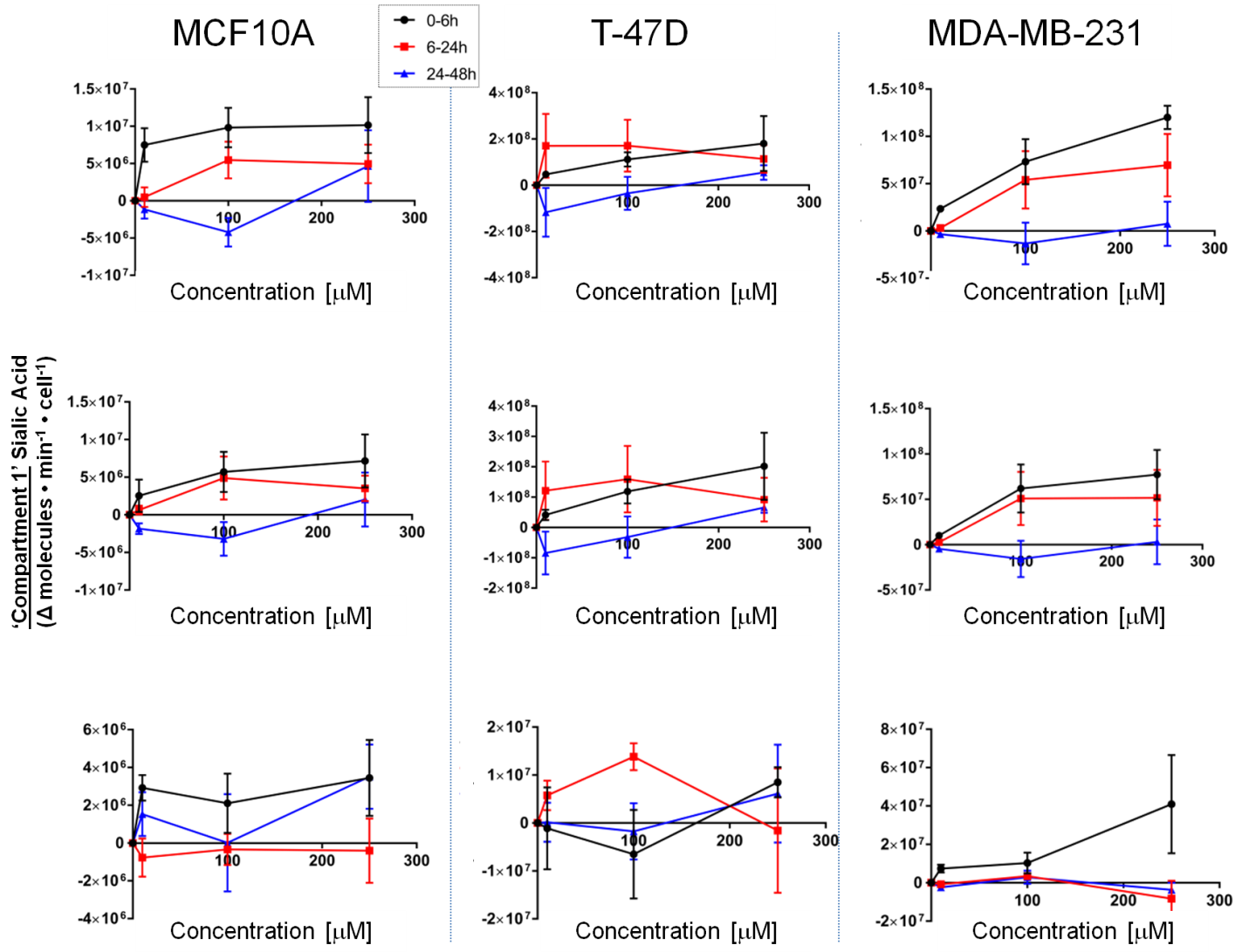


**(B) Rate of sialic acid production in 1,3,4-O-Bu_3_ManNAz-supplemented breast cell lines**:


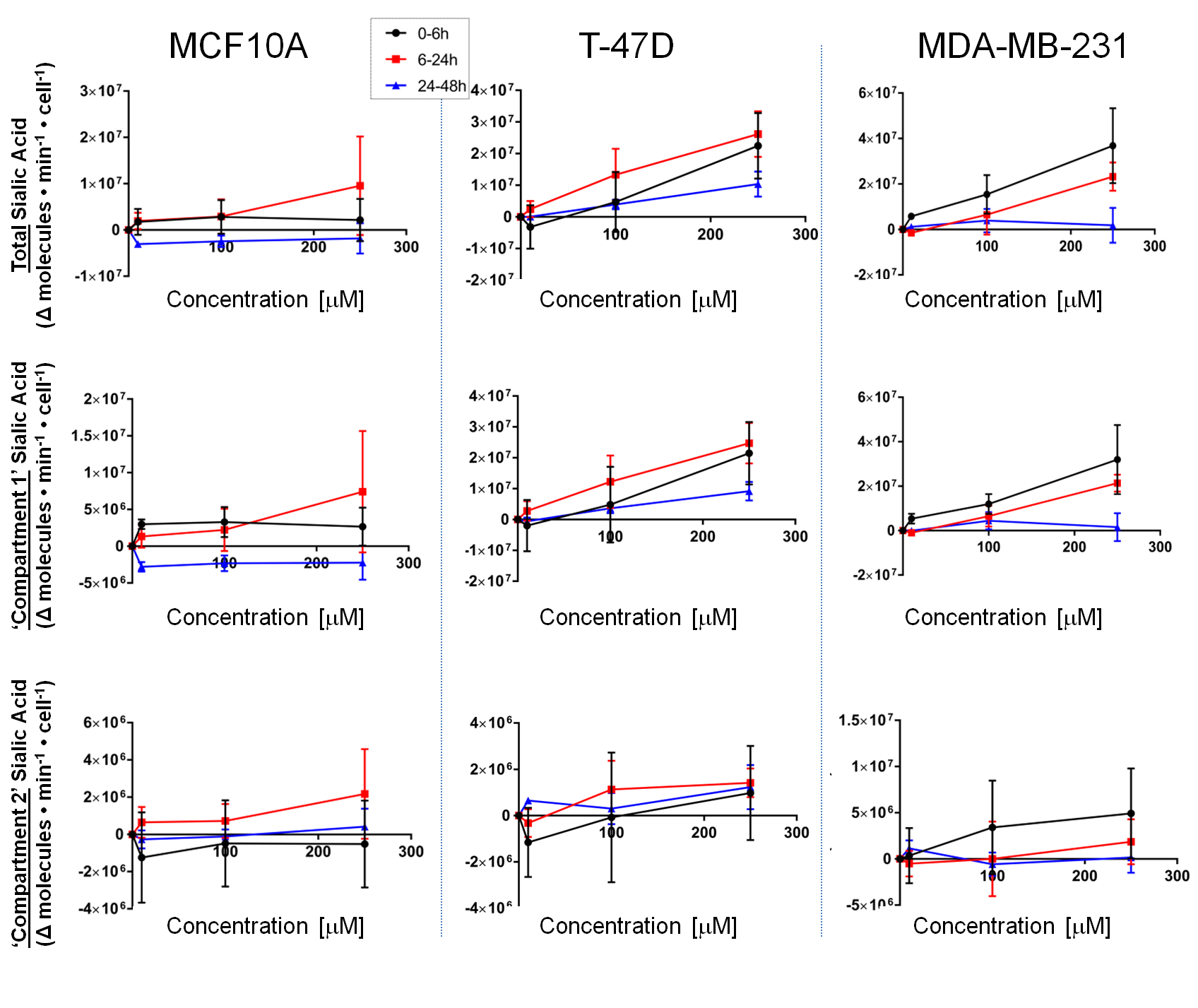


**(C) Rate of sialic acid production in 1,3,4-O-Bu_3_ManNAl-supplemented breast cell lines**:


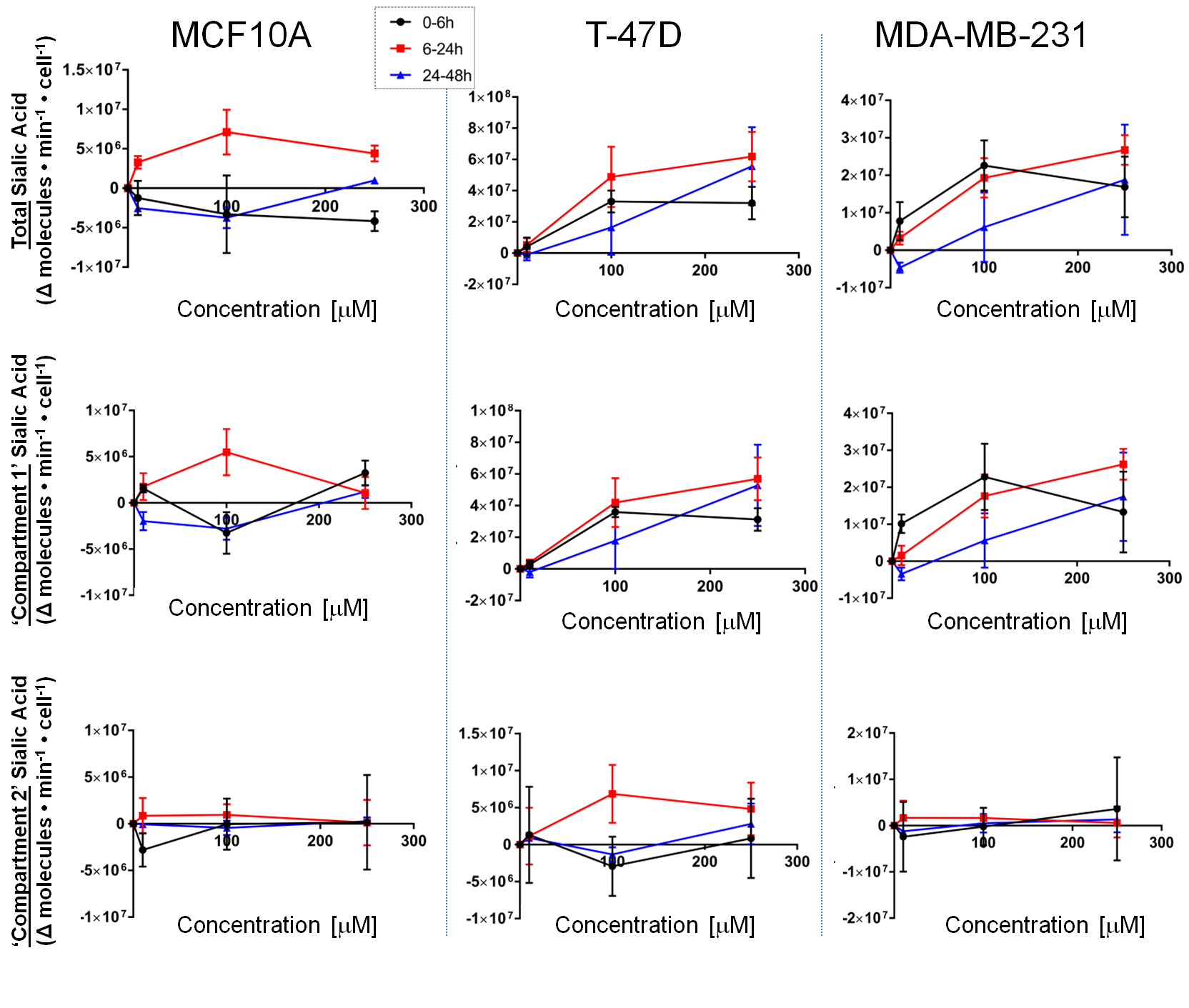

Supplement: S4 Fig — The change in the number of sialic acid molecules per cell per minute was calculated for each cell line (MCF10A, T-47D, and MDA-MB-231) for each cell line for the indicated time intervals after addition of 0, 10, 100, or 250 μM of each analog (1,3,4-O-Bu3ManNAc, 1,3,4-O-Bu3ManNAz, or 1,3,4-O-Bu3ManNAl) at time = 0 h. The rates of production (with negative values indicating a decrease in sialic acid during the indicated time interval) are shown in Panel A (this page) for 1,3,4-O-Bu3ManNAc, in Panel B (Page 9) for 1,3,4-O-Bu3ManNAz, and in Panel C for 1,3,4-O-Bu3ManNAl (Page 10). (DOCX) [file pone.0195812.s004.docx]
